# Computational meta-analysis of ribosomal RNA fragments: potential targets and interaction mechanisms

**DOI:** 10.1093/nar/gkab190

**Published:** 2021-03-27

**Authors:** Lingyu Guan, Andrey Grigoriev

**Affiliations:** Department of Biology, Center for Computational and Integrative Biology, Rutgers University, Camden, NJ 08102, USA; Department of Biology, Center for Computational and Integrative Biology, Rutgers University, Camden, NJ 08102, USA

## Abstract

The most abundant cellular RNA species, ribosomal RNA (rRNA), appears to be a source of massive amounts of non-randomly generated fragments. We found rRNA fragments (rRFs) in immunoprecipitated Argonaute (Ago-IP) complexes in human and mouse cells and in small RNA sequencing datasets. In human Ago1-IP, guanine-rich rRFs were preferentially cut in single-stranded regions of mature rRNAs between pyrimidines and adenosine, and non-randomly paired with cellular transcripts in crosslinked chimeras. Numerous identical rRFs were found in the cytoplasm and nucleus in mouse Ago2-IP. We report specific interaction motifs enriched in rRF-target pairs. Locations of such motifs on rRFs were compatible with the Ago structural features and patterns of the Ago-RNA crosslinking in both species. Strikingly, many of these motifs may bind to double-stranded regions on target RNAs, suggesting a potential pathway for regulating translation by unwinding mRNAs. Occurring on either end of rRFs and matching intronic, untranslated or coding regions in targets, such interaction sites extend the concept of microRNA seed regions. Targeting both borders of certain short introns, rRFs may be involved in their biogenesis or function, facilitated by Ago. Frequently dismissed as noise, rRFs are poised to greatly enrich the known functional spectrum of small RNA regulation.

## INTRODUCTION

A term ‘small RNA’ (sRNA) denotes a group of non-coding RNA (ncRNA) molecules typically <200 nucleotides (nts) in length. The most studied sRNAs include microRNAs (miRNAs) (1,2), small interfering RNAs (siRNAs) (3), PIWI-interacting RNAs (4), small nuclear RNAs (5) and small nucleolar RNAs (6). The best known of these, miRNAs, are loaded to Argonaute (Ago) complexes and act by preferentially binding 3′ untranslated regions (UTRs) of their target genes thus regulating mRNAs post-transcriptionally using 7-nt short ‘seed’ sequences (7).

Recent studies have significantly expanded the spectrum of sRNAs with the discovery of a broad repertoire of fragments generated from unexpected classes of RNAs with established ‘textbook’ functionalities, such as transfer RNAs (tRNAs) (8–17) and ribosomal RNAs (rRNAs) (18–22). RNA polymerase III transcribes the 5S rRNA and tRNAs and elevations of their levels are known as hallmarks of many malignant carcinomas (23–25). Recently, tRNA-derived fragments (tRFs) have been implicated in cancers and their signatures have been proposed as cancer biomarkers (26–28). Many studies have reported tRFs inhibiting protein synthesis in stress response (29–31) and suppressing cancer growth, cell invasion and metastasis (32). tRFs have been identified across all kingdoms of life and shown to have regulatory functions similar to miRNAs (8–11,14–16,33) or distinctly different from them (34). In fruit flies, multiple tRFs loaded to Ago have been suggested to function in a miRNA-like manner, given significant matches of their 7-mer ‘seeds’ to conserved 3′ UTRs of target mRNAs (16), consistent with experimental evidence for singular tRFs (11,12). Integrating sRNA- and RNA-Seq data from aging rat brain we have has shown that tRFs may induce comparable or even stronger effects than miRNAs on predicted target transcripts (15). Ribosomal proteins (RPs) have been reported among tRF targets across species, although with different effects. For example, a 3′ LeuCAG tRF may enhance the translation of RPS28 in mice and human and maintain the ribosome biogenesis (34), while in *Drosophila melanogaster*, tRFs have been shown to repress the RP expression and hence the global translation (35).

Such novel regulatory functionality in fragments of molecules with canonical roles motivated us to consider rRNA-derived fragments (rRFs), typically ignored in the sRNA analyses. rRFs have recently attracted significant interest but their exact roles are yet to be elucidated. rRFs have been found in multiple studies in plants, for example, originating from the 5′ end of a putative long form of 5.8S rRNA with precise cleavage site and tissue-specificity in *Piper nigrum*, with the homologous rRF differentially associated with Ago in *Arabidopsis* and rice (21). Rice calli express rRFs that map to 5.8S, 18S and 28S rRNA (22). In Chinese cabbage, heat-responsive rRFs are produced primarily from the 3′ end of chloroplast 4.5S and 5S rRNAs (36). In *Drosophila*, a non-canonical sRNA conserved across seven fly species has been identified within the 45S precursor rRNA (45S pre-rRNA) internal transcribed spacer (20). In *Amblyomma testudinarium*, rRFs from the 5′ and 3′ ends of 5.8S and 28S rRNA have been reported, with a significantly higher abundance compared to rRFs from the middle of these rRNA molecules (22). In murine embryonic stem cells (mESC), rRFs from mature 28S and 18S rRNAs have shown enrichment in P19 protein, known to bind siRNA-like double-stranded RNAs of 19 nt in length (37). The cleavage of rRNA by angiogenin (ANG) has been long known (38) and may be relevant for the rRF formation. Sequencing of human sRNAs containing 2′,3′-cyclic phosphate has detected rRFs produced from nuclear rRNAs under stress conditions (39), pointing to a potential cleavage by ANG or other endoribonucleases. A recent paper has reported rRFs in human lymphoblastoid cell lines samples (40) and linked variation of the rRF expression with ethnicity, sex and tissue.

However, despite this accumulating evidence for rRFs in multiple organisms, their functional roles remain unclear. The involvement of 5.8S rRF in the cleavage of plant RPS13 and RPL5P mRNAs has been demonstrated in black pepper and the *Arabidopsis* degradome data (21). A knockdown of a 20-nt rRF in H1299 cells induced apoptosis, inhibited cell proliferation and led to a significant decrease in G2 phase cells (22). A study in HeLa cells has shown an inhibition of several RPs caused by overexpressing a rRF from the 5′ end of 28S rRNA (41). Age-associated rRF changes in different Ago proteins have also been shown in *Drosophila* (42). While growing in number, such isolated cases of functional analysis have not yet painted a consistent picture of the role of rRFs, underscoring the need for a comprehensive study of these potential novel regulators.

**Figure 1.**
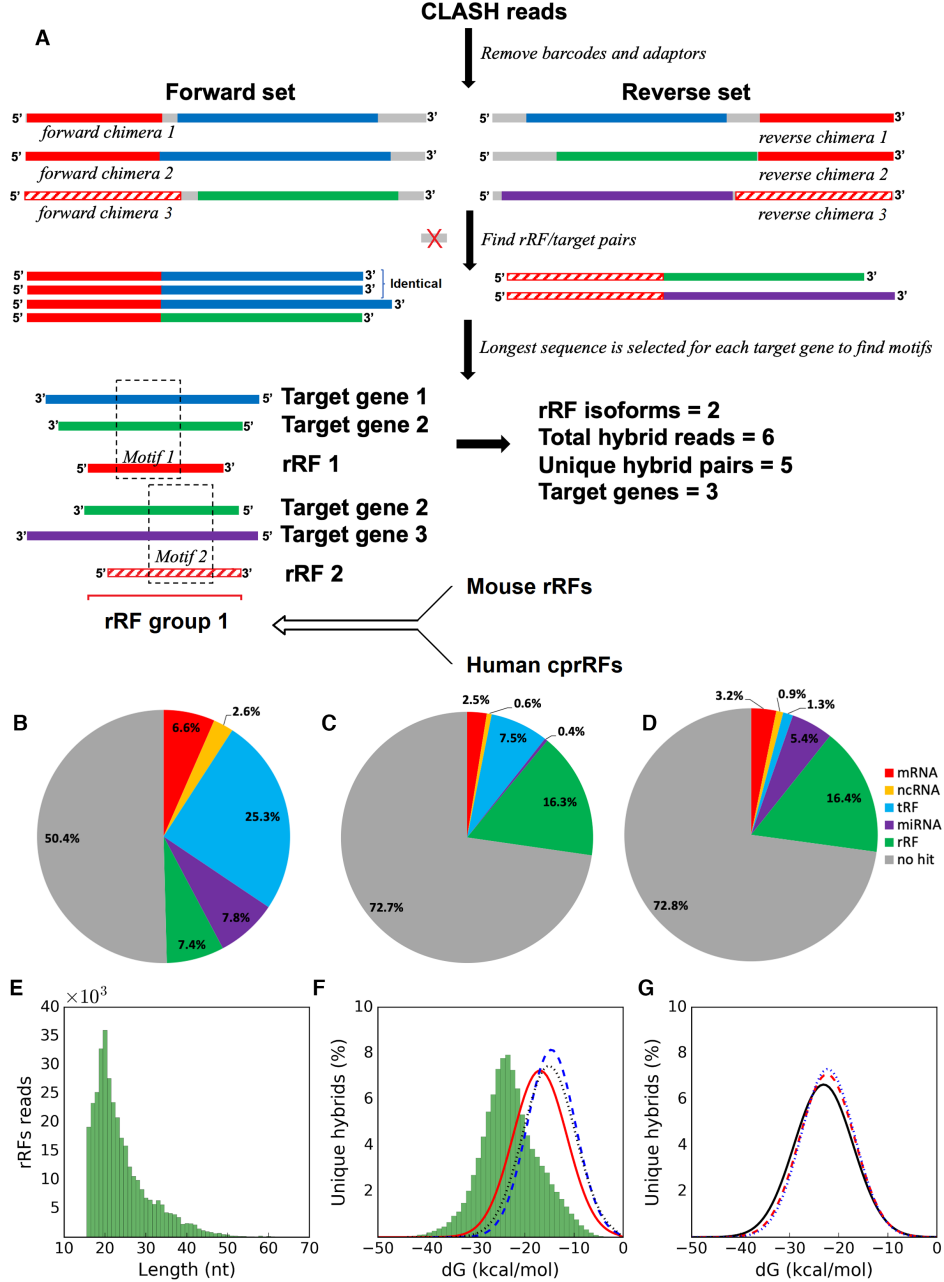
Analysis and properties of rRFs and other sRNA guides paired with targets in CLASH hybrids. (**A**) Overview of the meta-analysis of different datasets. Boxes of the same color in CLASH chimeras (top) correspond to the same rRF isoforms or fragments of the same target gene. Gray boxes are nucleotides not matched to rRNA or a target gene. A group (bottom) is produced from overlapping rRFs containing motifs (see section E and Methods) and information from other datasets is integrated with the group to produce a final summary (Supplementary Figure S8). Many more reads (∼10^6^) and unique hybrids (∼10^5^) are typically found and only a few are shown for clarity. Pie-charts show the distributions of various types of targets paired with (**B**) rRFs, (**C**) miRNAs and (**D**) tRFs. Paired target types are color-coded in (B–D), for example, 2.5% of all CLASH targets paired with miRNA guides represent mRNAs (C). Sometimes guide-guide pairs were seen, for example, 0.4% of all CLASH targets paired with miRNA guides represent other miRNAs (C). See Methods for details of the target type assignment. (E) Length distribution of all rRFs identified from CLASH chimeric reads. (**F**) The MFE distributions of shuffled rRF-target pairs (red solid line) as well as miRNAs (blue dashed line) or tRFs (black dotted line) artificially paired with rRF targets represent randomized controls. The much stronger interactions of specific rRF-target pairs (non-redundant) that we derived from CLASH chimeras are shown as the green histogram. (**G**) The MFE distributions of rRF-mRNA pairs (black solid line), rRF-tRF pairs (red dashed line) and rRF-miRNA pairs (blue dotted line).

Here, we investigated the mode of action of rRFs using a computational meta-analysis involving a number of large-scale experimental datasets of sRNA in human and mouse cells (Figure 1A). In human, we analyzed the data of a method capturing *in vivo* RNA–RNA interactions as sRNA–mRNA chimeric sequences by Crosslinking, Ligation, and Sequencing of Hybrids (CLASH). CLASH experiments, with multiple library replicates showing consistent patterns, have been developed to study miRNAs by directly connecting them with their targets (43,44) and also used to identify tRFs as potential miRNA-like regulators (14). We have recently demonstrated the value that can be extracted from the CLASH dataset when studying the patterns of interaction of tRFs with their targets, suggesting novel binding motifs and interaction mechanisms (45). We have predicted interacting regions for two dozen tRFs, validated by matches to their motifs in many cases, where such regions have been determined experimentally (46,47). We applied this methodology here and predicted 680 rRF motifs that may drive interactions with thousands of protein coding targets. In the Ago-IP of cytoplasmic and nuclear fractions of two different murine cells, we also detected rRFs largely overlapping human CLASH rRFs.

This meta-analysis allowed us to generate a number of striking observations and intriguing hypotheses. However, as a clarification, we use the words ‘target’, ‘binding’ and ‘interaction’ for short in this paper to call *putative* targets, binding and interactions, as this is a bioinformatics study and our results are inferences on prior experiments. While aiming to isolate sRNA-target pairs directly loaded to Ago, CLASH might detect abundant but unrelated RNAs. Hence, we extensively analyzed available data from other crosslinking and immunoprecipitation (CLIP) and sRNA-Seq experiments in human and mouse cells for additional evidence.

We performed a number of statistical tests to check if rRFs may result from a random rRNA degradation or are a byproduct of rRNA maturation processes. We observed a non-random pairing of rRFs to potential target RNAs in CLASH and noted significant nucleotide content biases at rRF borders and in the likely regions of target binding. Our analysis of intra-molecular secondary structures of mRNA in HEK293 cells (48) showed that such targeted mRNA regions had striking preferences for being double-stranded, similar to a recently validated tRF-targeted site on RPS28 mRNA (34). These interacting regions were also spatially compatible with the Ago crosslinking patterns we found in PAR-CLIP (Photoactivatable-Ribonucleoside-Enhanced Crosslinking and Immunoprecipitation) datasets in human (49) and mouse (50). We observed that rRFs may bind not only mature mRNAs (3′UTR > CDS > 5′UTR) but also intronic regions of various genes. We noted that a group of short introns, agotrons, previously identified as Ago-associated regulators (51), was often targeted by rRFs precisely at either the 5′ donor site or 3′ acceptor site. Finally, we considered scenarios of random and non-specific rRF binding to Ago and putative targets and proposed potential mechanisms for the generation and possible functionality of rRF. Our computational meta-analysis opens the way for systematic experimental testing of the predicted interaction sites and potential mechanisms of these novel potential regulators, originating from the most abundant RNA species in every living cell.

## MATERIALS AND METHODS

### Analysis of CLASH data

Throughout the paper, we used scipy 0.16.1 for statistical tests and *P* < 1E–16 indicates the *P*-value is smaller than the minimum positive float number. CLASH data for HEK293 cells (44) were downloaded from the SRA database (SRR959751 to SRR959759). We used Fastx_toolkit 0.0.13 (http://hannonlab.cshl.edu/fastx_toolkit/) to remove barcode and adapter sequences and collapse identical reads. We used an in-house developed computational pipeline to analyze different guide RNAs (miRNAs, tRFs and rRFs) in a consistent way. We first ran Bowtie 2.2.5 (local mode) to align all reads to rRNAs, tRNAs and miRNA genes, and reads with ≥ 80% alignment matches (M in CIGAR string) were removed from further analysis as not having a target in the read. We then identified all guide RNAs from hybrid reads, allowing no mismatch and giving preference to longer guide isoforms. In detail, the aligner determined if a hybrid read started with a known guide host sequence (miRNA from miRbase (52), tRNA from GtRNAdb and tRNAdb (53,54) and rRNA genes from RefSeq (https://www.ncbi.nlm.nih.gov/refseq/) and Ensembl (55) and checked if the next nucleotide still belonged to the same host sequence, stopping at the first mismatch. To identify guides on the other end of a hybrid read, we used the same aligner and searched for matches in the opposite direction starting from the 3′ end of the read. The longest matched isoform was identified as the guide sequence and the remainder of the hybrid read was considered a targeted sequence. Targeted sequences shorter than 20% of the read length were excluded. Each target was first checked in the human transcriptome (Ensembl 91) using BLAST (word size 7, default scoring matrix, *e*-value < 0.1). Targets that could not be mapped to the transcripts were then aligned to the human genome (hg38) using BLAST. Reads were considered as chimeras if the combined length of rRF and targeted sequences was ≥75% of the total length of the hybrid read after adaptor and barcode clipping. rRF-containing chimeras with targeted sequence that mapped in the same orientation as transcription were considered for downstream analysis. To obtain high-confidence mRNA targets, we checked if BLAST alignments of such targets to miRNA, tRNA or rRNA sequences showed >90% identity. If so, they were removed from the list of mRNA targets and also from guide RNAs if they had mismatches with respective guide host genes. If a target sequence was aligned to multiple transcripts of one gene with same e-value, we gave preference to the protein coding transcript. Each guide-target pair was counted only once when analyzing the distribution for target RNA biotypes and targeted regions. In the following sections only rRF guides were analyzed, unless otherwise specified.

### Analysis of PAR-CLIP data, sRNA sequencing data and icSHAPE data

High-throughput sequencing datasets for Ago1 to Ago4 PAR-CLIP in HEK293 cells (49) were downloaded from SRA database (SRR048973 to SRR048979) and processed as above. We used Bowtie 1.0.1 (56) to align the reads to rRNA references in end-to-end mode, allowing one T to C mismatch and giving preference to perfect matches, as in an earlier tRF analysis (14). rRFs shorter than 16nt were excluded. The abundance of each rRF isoform was normalized to reads per million mapped to the human genome (RPM). We used mismatches identified in PAR-CLIP reads and rRNA sequences as T→C conversion sites and mapped them to positions in CLASH rRFs. PAR-CLIP dataset for mouse Ago2 (50) was analyzed using the same pipeline. Mouse rRNA sequences were aligned to human rRNAs using clustalw (https://www.genome.jp/tools-bin/clustalw) to match T→C conversion sites from mouse rRFs to human rRFs. The sRNA sequencing datasets used in Supplementary Table S8 were downloaded from SRA and GEO using accession numbers shown in the table. We analyzed each library using same pipeline as for PAR-CLIP but allowing no mismatches. 18S and 28S 5′ terminal rRFs were defined as the isoforms covering the first 5nt of mature rRNAs.

icSHAPE reactivity scores for cytoplasmic RNAs in living HEK293 cells were downloaded from the UCSC genome browser (48). We collected the scores for each nucleotide position in the target sequence identified in CLASH chimeric reads and in the flanking sequence. Target sequences without valid icSHAPE scores (such as introns) were excluded from analysis.

### Analysis of secondary structure of rRFs and targets and their binding energy

We downloaded the *cis* Watson-Crick base pairing information for rRNAs from RiboVision website (http://apollo.chemistry.gatech.edu/RiboVision/). All of the rRFs identified in CLASH chimeric reads were aligned to the rRNA sequences provided by RiboVision to determine whether their boundaries were in paired region or not. The coordinates of expansion segments (ES) were obtained from (57) and were aligned to the reference rRNA sequences that were used for finding the rRFs. rRF was considered overlapping a given ES if it shared at least 10 nt with the ES or covered shorter ES completely. We used RNAhybrid 2.1.2 (58) with default parameters to predict the secondary structure of each rRF-target interaction identified from CLASH data. The minimum free energy (MFE) of predicted structure was used to evaluate the binding intensity between rRF and matching target. As comparison, we introduced two control groups: (i) pairs of shuffled rRFs and rRF targets or (ii) random pairs of miRNAs or tRFs identified in CLASH data and rRF targets. We compared the MFE distribution between genuine rRF-target interactions to control groups and estimated the significance using two-tailed Student's t-test. The secondary structure of mRNA was predicted by RNAfold (59) and visualized in VARNA (60).

### Analysis of rRF binding patterns and motifs

We followed our earlier approach (15,16) to investigate rRF-target hybridization patterns. We selected all unique chimeras formed by protein coding targets and 20-nt rRFs from 18S rRNA (or 28S rRNA). The rRF-target pairs were further collapsed to minimize potential bias caused by duplicated hybrids: we considered chimeras with targets differing by <5nt in length at both ends as identical (i.e. a target overhang of five or more bases was considered a different chimera). In the predicted rRF-target secondary structures we encoded each nucleotide of the rRF as 1 (if bound to the target) or 0 (if not) allowing G:U pairs. As a result, we obtained a data frame with 20 binary features and used *scikit-learn* (61) to perform unsupervised clustering using k-means method.

We then looked for potential binding motifs on rRFs. For one rRF isoform, we used the longest sequence detected for each targeted gene in the CLASH chimeric reads with this isoform. We used MEME (62) to search motifs in these target sequences with the default parameters and also with the equifrequent background model, typically obtaining overlapping motifs for both cases. Target motifs with e-value < 0.01 were included. We used FIMO (63) to find the best complementary match on the rRF sequence (with *P*-value < 0.001). After this, a rRF-target pair was deemed to contain a motif if the MEME position *P*-value of the target was <0.05. Overlapping rRFs were binned together to form a group. For each group, we generated combined plots of T→C conversions, rRF-target binding from RNAhybrid, logos of relative frequencies of rRF nucleotides in motifs and multiple statistics as shown in Supplementary Figure S8.

We selected a high confidence set of targets as those passing two thresholds, on unique hybrids (UH) and read counts (RC). We found a target supported by the highest number of unique hybrid pairs (for UH) and a target supported by the highest number of reads (for RC) and used 1% of each of these numbers for a corresponding threshold. For KEGG pathway enrichment pathfindR (64) was run on target genes, using artificial *P*-values of 0.05 for each. For Gene Ontology analysis we used http://geneontology.org, with website's default parameters.

## RESULTS AND DISCUSSION

### A. rRFs are likely non-random products primarily originating from mature rRNAs and are paired with non-random sequences in numerous CLASH chimeras

We analyzed CLASH data for HEK293 cells (44) to examine *in vivo* formed chimeras between small RNAs (guides) and their respective targets. In addition to miRNAs and tRFs, reported earlier (14,44,45), we observed rRFs as guides in CLASH chimeras, with 2-fold frequency of miRNAs and two-thirds of tRFs (Supplementary Table S1). Unlike previous analyses of this CLASH dataset, which have focused on just one guide type (miRNAs or tRFs), we first considered all guides together and evaluated their propensities of forming chimeras. We found that guide-guide chimeras and unpaired guides dominated CLASH reads (Figure 1B-D). Intuitively, this would be expected if guide-loaded Ago1 is in excess and the efficacy of guide-target ligation is low in CLASH. Other explanations would involve potential guide-guide interactions at a scale much greater than those between sRNA and mRNA (Supplementary Table S1, Figure 1B–D, see section C).

In this dataset, rRFs had the lowest share of reads without any targets and highest – paired with mRNA or non-guide ncRNA (9.2% versus 4.1% for miRNAs or 3.1% for tRFs, Figure 1B–D, Supplementary Table S1). Thus, CLASH appeared as suitable for studying potential rRF targets as for the other guide sRNAs (14,44,45). Here, we call chimeras forward or reverse, if guides were found on their 5′ or 3′ end, respectively (Figure 1A). Properties of the rRF guides and their targets were generally very similar in both forward and reverse orientations (see section C). We compared guides starting at the first three positions in forward chimeras or ending at the last three positions in reverse. As nearly all of the guides started or ended at the exact 5′ or 3′ read terminus (97% of rRFs), we only considered such reads in further analysis, with no mismatches for guide host genes.

The vast majority of CLASH rRFs were 16–25 nt long, with the most frequent length of 20 nt (Figure 1E). The narrow length range resembled those seen in miRNA and tRFs suggesting that there may be specific rules, according to which rRNAs are cleaved into distinct isoforms. This distribution may indicate either a functionally required size, or a length protected by Ago, preventing rRFs from degradation. Given this duality, we asked if rRFs were produced by directed cleavage or by random degradation using a number of tests throughout this study. The CLASH procedure may cause certain biases and a possible degradation of the RNA ends. However, our focus here is on potential binding regions, likely protected by Ago, and their properties should remain intact.

**Figure 2.**
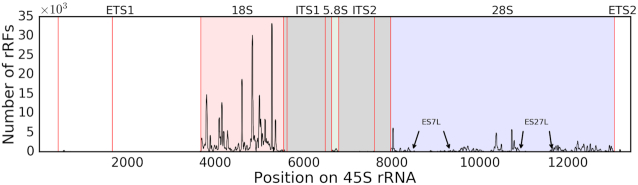
CLASH rRFs originated from multiple regions across 45S pre-rRNA. The number of identified rRFs covering every nucleotide of human 45S pre-rRNA (GenBank U13369.1) is plotted along the length of 45S. Pale red, green and blue boxes correspond to the 18S, 5.8S and 28S mature rRNAs, respectively. Gray boxes correspond to the ITS domains. Red vertical lines indicate the endonucleolytic cleavage sites (92). Arrows indicate borders of two largest expansion segments in 28S rRNAs, absent from *E. coli* and present in human (57).

Random mechanisms of rRF generation would logically suggest randomness in pairing of rRFs and their targets in CLASH chimeras, and vice versa. To test this, we calculated the minimum free energy (MFE) of hybridization for each rRF-target chimeric pair using RNAhybrid (58). We found that predicted MFE of rRFs bound to their targets was significantly lower (Figure 1F) compared to the shuffled pairs of rRF and targets (*P* < 1E–16) and to pairs of random (miRNA or tRF) guides with random rRF targets (*P* < 1E–16). The MFE distributions were similar for all guides, but rRFs showed a smooth curve with the lowest number of extra peaks when paired with mRNA or ncRNA targets (Supplementary Figure S1). The rRF dataset seemed to contain less noise than other guides and had a similar distribution of chimeric reads. This supported a likely non-random, interaction-driven pairing of rRFs and their specific targets in CLASH chimeras. We note that ∼99.5% of the CLASH rRFs from 45S pre-rRNA were contained within mature rRNA borders (Figure 2). This argues against random breakage of the pre-rRNA as the source of rRFs and is consistent with the results in numerous non-Ago samples (40). We tested whether the flanking regions of the rRNA genes or their antisense strands contributed to the rRF pool found in CLASH and found negligible amounts of reads aligned to the antisense rRNAs (0.28%) and the upstream flanking regions of rRNA genes (0.01%). The 3′ flanking regions of the 5S rRNA produced 1.2% of rRFs.

### B. rRFs originate from specific positions across rRNA molecules, with starts and ends predominantly in single-stranded regions

The rRF profile across the 45S pre-rRNA revealed a highly non-uniform pattern (Figure 2), with the 18S rRNA hosting the most abundant rRFs. They originated from multiple positions on the 18S rRNA, although several distinct peaks gave rise to the overwhelming majority (>80%) of the CLASH rRFs. In contrast, other mature rRNAs showed different fragmentation patterns. For example, rRFs mapped primarily to the 5′ arm of the 5.8S rRNA (Figure 2) but the 3′ arm of the 5S rRNA (data not shown). rRFs were scattered across the 28S rRNA with only a few prominent rRF-rich sites (Figure 2). ETS (External) and ITS (Internal transcribed spacer) regions had <0.6% of all CLASH rRFs.

We observed that rRFs started and ended in single-stranded (SS) loops significantly more frequently than expected and significantly more frequently than in double-stranded (DS) helices (Table 1). The secondary structure is not well defined for some large rRNA insertions, known as expansion segments (ES), which are GC-rich in human and are seen as flexible tentacle structures in electron microscopy images (57). Excluding the two largest ES, 7L and 27L, we observed an even stronger preference for both rRF ends in SS regions of 28S rRNA (Table 1). For every rRNA, the largest populations of rRFs had SS-only and SS-DS ends. This indicated an overall non-random distribution of rRF ends across the whole mature rRNAs but also pointed to a likely breakage (or endoribonuclease digestion) in the less stable SS regions (see section D).

**Table 1.** Boundaries of rRFs in double-stranded (DS) or single-stranded (SS) regions of rRNA

		rRFs with both boundaries in SS region		rRFs with both boundaries in DS region	
rRNA type	Number of DS nts/length	Expected	Observed	Expected	Observed
5.8S	82/157	22.82%	47.85%*	27.28%	10.08%*
18S	908/1869	26.44%	44.81%*	23.60%	10.46%*
28S	2082/5070	***34.73%***	36.14%*	***16.86%***	15.04%*
28S (excluding ES7L and ES27L)	1738/3476	25.00%	38.43%*	25.00%	14.66%*
5S	72/121	16.40%	22.09%*	35.41%	19.38%*

* Significant at *P*-value < 1E–16 (Chi-square test).

We observed no obvious link of the rRNA accessibility with frequency of rRF CLASH reads. For example, hairpins 39–40 and 42 in 18S, corresponding to the regions of lowest accessibility in *E. coli* 16S (65), produced abundant rRFs. The ES insertions increased the size of rRNA from prokaryotes to eukaryotes (57), they protrude from the ribosome and are accessible. However, the number of rRFs overlapping known human ES by ≥10 nt (Supplementary Table S2) showed the largest ES, 27L, was practically devoid of rRFs and with low density in other large segments (Figure 2, Supplementary Table S2, Supplementary Figure S2), an unlikely outcome if rRFs were generated randomly or primarily from accessible rRNA regions. In contrast, rRFs were mainly formed from the rRNA core regions. Such depletion indicates that either composition, function or accessibility of the large ES prevents them from generating rRFs. Another intriguing explanation is that the rRFs may be conserved from the earlier organisms, whose rRNAs lacked the ES. This is consistent with the enrichment of rRF targets in fundamental metabolic and information processing pathways (see section G).

There were intriguing exceptions among the short ES, in line with the non-randomness. For example, a 5-nt long ES14S in 18S had the highest density of rRFs/nt (Supplementary Table S2). Two rRFs from a small ES7S may be involved in a potentially complex interaction with the mRNA of RPS28 (discussed in section G). Could structural features of some short ES be recognized (e.g. by endoribonucleases) and specifically cut to produce rRFs?

### C. In both CLASH orientations, rRFs pair with various target types, the largest target group comprising exons and introns of protein-coding genes

There are very limited datasets with guide-target pairs, hence we attempted to extract maximum information by including all possible CLASH interactions. We also constructed a high confidence subset of interactions with stringent requirements (see section E). We identified a total of >2.2 million rRF-target reads, with an astounding 46% of them—in reverse pairs with tRFs (Table 2). This large bias was mostly due to a handful of tRFs (ArgTCG > IleTAT >AlaAGC > LeuTAA > CysGCA), which were very different from dominant tRFs (AspGTC >> GluCTC > GlyGCC) in the forward orientation (Supplementary Table S3). Pairing of rRFs and miRNAs also showed strong bias. For example, ∼52 000 forward chimeras contained mir-92a, in >40% cases pairing with almost identical rRFs 18S-1446–1463 and 18S-1446–1464 (this notation denotes each rRF as ***host_rRNA-start-end***). In reverse, >50% miRNA-rRF reads revealed pairing of mir-125 and multiple rRFs (Supplementary Table S3) including 18S-1598–1616, 18S-1153–1172, etc.

**Table 2.** Numbers of interactions between rRFs and different target types

	Target	Unique hybrids	Total reads
**A**	mRNA	166 653 (60 497)	348 029 (241 873)
	tRF	131 573 (67 020)	1 339 603 (1 275 050)
	miRNA	45 325 (24 032)	414 151 (392 858)
	ncRNA	57 052 (21 941)	137 361 (102 250)
	Total	400 603 (173 490)	2 239 144 (2 012 031)
**B**	mRNA	79 552 (26 493)	170 569 (117 510)
	tRF	56 239 (28 951)	306 488 (279 200)
	miRNA	22 270 (12 265)	199 362 (189 357)
	ncRNA	24 070 (8243)	56 026 (40 199)
	Total	182 131 (75 952)	732 445 (626 266)
**C**	mRNA	87 214 (34 038)	177 460 (124 284)
	tRF	78 048 (39 298)	1 033 115 (994 365)
	miRNA	23 948 (12 175)	214 789 (203 016)
	ncRNA	33 123 (13 734)	81 335 (61 946)
	Total	222 333 (99 245)	1 506 699 (1 383 611)

Numbers in parenthesis are rRF-target interactions supported by at least two CLASH chimeric reads. (A) Union of forward and reverse pairs, (B) forward pairs and (C) reverse pairs. Since some unique pairs are the same in B and C, their sum is greater than the value in A.

However, when we considered only unique guide-target pairs, the totals above collapsed ∼400 000 rRF-target chimeras and protein-coding targets comprised the largest (>41%) group of unique chimeras (Table 2). Furthermore, the binding of rRFs and mRNA targets was also the strongest (Figure 1G), compared to the rRF hybrids with miRNAs and tRNAs. Similar energetically (Supplementary Figure S1), rRF–mRNA chimeras outnumbered rRF-ncRNA pairs (Supplementary Table S1), including long ncRNAs, RNA Y, vault RNA, pseudogenes and those annotated only as ‘misc RNAs’. Since both guide-target orientations appeared possible in CLASH, we could not unequivocally identify a target in guide-guide interactions. The roles of other ncRNA are typically even less clear and may represent a challenge in the interpretation. Furthermore, while overabundant binding of a few distinct guides may indicate a biological signal, it does not help with identifying binding patterns, which require a diverse set of target sequences. Hence, for this first study of potential mechanisms of rRF-target binding we chose a reductionist approach, leaving potentially complex networks of guide-guide interactions (which may also represent possible experimental artifacts) aside.

**Figure 3.**
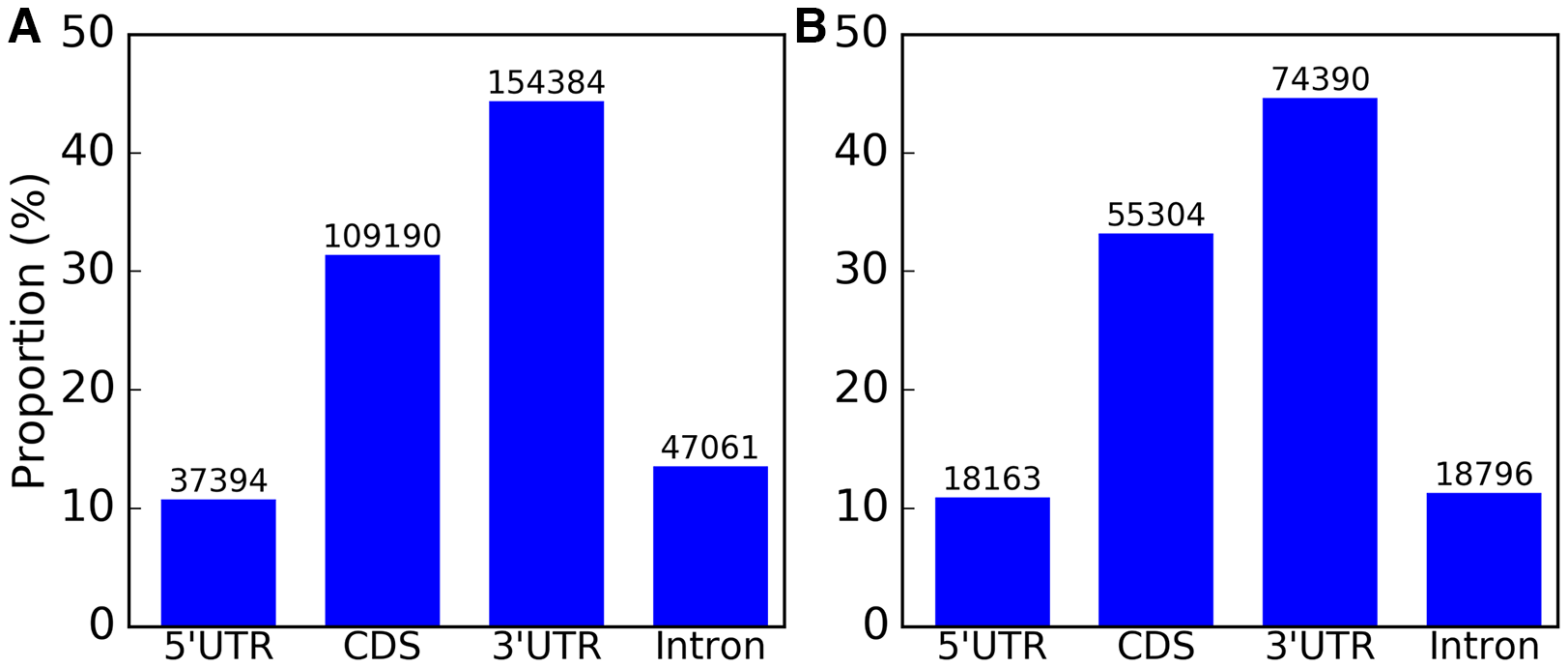
Distribution of putative rRF–targeted mRNA regions. Similar distributions are seen for (**A**) all rRF–mRNA chimeric reads sequenced in CLASH and (**B**) unique rRF–mRNA hybrid pairs.

We thus limited our further analysis to transcripts of protein coding genes, following the traditional studies of sRNA targets. 18S and 28S rRFs interacting with mRNA had a peak length of 20 nt, showing a much greater abundance compared to other rRFs (Supplementary Figure S3). We found that untranslated regions (UTR) were targeted most frequently (with the same distribution of targeted regions for all sequenced chimeric reads and collapsed unique chimeras, Figure 3). The targeting rate was similar to previous findings for miRNAs and tRFs (44,45,49,66,67). It was ∼4-fold higher for 3′ than 5′ UTR. CDS targeting frequency was three times that of 5′ UTR. Similar to tRFs (45), >12% of rRF interactions with protein coding transcripts occurred in the introns.

Earlier, we have described that tRFs potentially interact (45) with the 5′ border of a specific group of short introns, ‘agotrons’. Agotrons have been previously detected in Ago2-IP libraries as new species of putative RNA regulators (51), but also argued to be protecting Ago from loading RNA degradation products to eliminate aberrant regulation (68). We found that 30 reported agotrons (51) can be targeted by rRFs (Supplementary Table S4). Except for ENO3, FAM129B and INTS1, all agotrons interacted with rRFs close to exon-intron borders (0–5nt) and in 18 genes exactly on the border. Many were targeted by numerous rRFs and, as noted earlier (45), by tRFs and miRNAs. We found ten agotrons with both 5′ and 3′ ends interacting with rRFs (Supplementary Table S4), and several rRFs with many targets (e.g. 18S-1678–1694 pairing with five agotrons). We further investigated all intron targets of rRFs and identified 384 genes with additional agotron–intron candidates (shorter than 200nt and interacting with rRFs within 5nt from either 5′ or 3′ border) for future experimental validation (Supplementary Table S5). Chimeras of unusual introns with significant enrichment of rRFs right on their borders (chi-square *P* < 1E–16) provided another indication of non-randomness of rRF-target pairing in CLASH reads, similar to the pairing of tRFs/miRNAs with agotrons.

**Figure 4.**
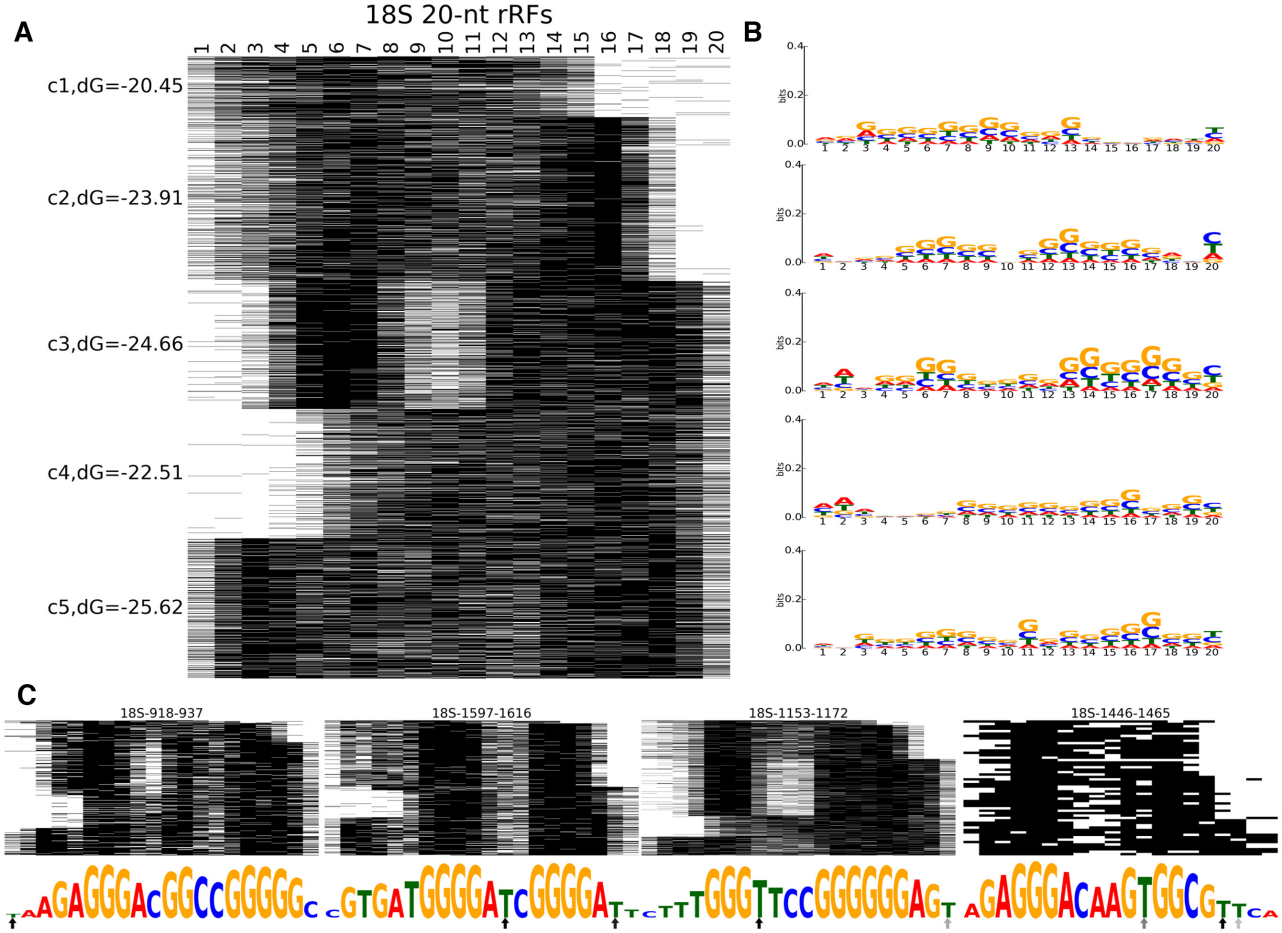
Clustering of 20-nt rRFs from 18S rRNAs reveals potential binding modes. (**A**) Base-pairing patterns of the 20-nt rRFs in 4603 unique rRF-target interactions. (**B**) Sequence logos show the nucleotide composition patterns for unique rRF isoforms in every cluster. (**C**) Base-pairing patterns of four 18S rRFs with the most interactions with different target genes. Sequence logos below show the frequency of hybridization with the target at each rRF position. The arrows show the frequency (darker shade being more frequent) of T→C conversions in PAR-CLIP data (see section E).

Such interaction sites of small RNAs located very close to or just on the agotron border indicated a mechanism beyond a simple competition for Ago loading (Supplementary Table S4). These agotrons we identified as rRF targets have G-rich downstream of the 5′ donor site and C-rich upstream of the 3′ acceptor site. Earlier, we have speculated that splicing of these introns may be facilitated by sRNAs (45), as the unusual 3′ C-richness may affect the U2 snRNP auxiliary factor (U2AF65) binding in the splicing process (69), but we have not seen tRFs directly interacting with the 3′ end of the agotrons in that study. Here, we observed multiple examples of rRFs, sometimes involving additional sRNAs, binding to both 5′ and (much more frequently) 3′ end of the agotrons (Supplementary Tables S4-S5). The G-richness of rRFs sequence (Figure 4) may be a factor that discriminates rRFs from tRFs (G versus C-rich in binding regions, Table 3) in targeting the C-rich 3′ end of agotrons. Overall, it strengthens our earlier hypothesis (45) that sRNAs may (i) play a role in splicing of agotrons or (ii) be bound by agotrons after splicing (functionally or in a sponge-like manner with their G- and C-rich ends), which is interesting given the proposed regulatory role of agotrons (51).

**Table 3.** Nucleotide contents of analyzed RNAs

	Position type	Adenosine (%)	Guanosine (%)	Cytidine (%)	Uridine (%)
**A**	All rRF positions paired with mRNAs	14.18	47.28	21.05	17.49
	All rRF positions unpaired with mRNAs	28.23	21.23	23.57	26.97
	18S 20-nt rRFs paired	12.26	57.15	15.25	15.34
	18S 20-nt rRFs unpaired	22.49	19.36	24.03	34.12
	28S 20-nt rRFs paired	17.67	45.91	21.36	15.05
	28S 20-nt rRFs unpaired	36.51	18.65	23.17	21.67
**B**	45S rRNA	12.09	36.87	35.51	15.53
	18S rRNA	22.39	29.45	26.67	21.49
	28S rRNA	15.89	35.97	33.21	14.95
	5.8S rRNA	19.75	28.66	28.66	22.93
	5S rRNA	25.22	26.95	23.07	24.76
**C**	miRNAs paired with mRNAs	14.74	31.76	27.49	26.01
	miRNAs unpaired with mRNAs	27.01	18.87	22.48	31.64
	tRFs paired with mRNAs	9.85	28.39	42.05	19.71
	tRFs unpaired with mRNAs	23.22	18.53	31.22	27.03
**D**	rRF 5′ end nucleotide	**38.01**	18.03	21.38	**22.58**
	rRF 3′ end nucleotide	15.06	9.66	***34.83***	***40.46***
	Upstream nucleotide flanking rRF	8.39	21.83	31.59	38.19
	Downstream nucleotide flanking rRF	42.39	24.93	18.37	14.3
**E**	miRNA 5′ end nucleotide	**23.22**	9.98	19	**47.79**
	miRNA 3′ end nucleotide	***20.78***	19.59	16.33	***43.3***
	Upstream nucleotide flanking miRNA	14.55	43.51	26.68	15.27
	Downstream nucleotide flanking miRNA	15.9	26.59	17.76	39.75
	tRF 5′ end nucleotide	**34.05**	9.38	8.83	**47.74**
	tRF 3′ end nucleotide	***51.38***	6.06	***23.77***	18.79
	Upstream nucleotide flanking tRF	67.95	11.52	10.72	9.81
	Downstream nucleotide flanking tRF	33.44	14.52	31.43	20.61

(A–C) Nucleotide compositions of guide sRNAs in the regions paired or unpaired with target mRNAs and in the host rRNA genes. (D–E) Terminal/flanking nucleotides in guide sRNAs. Top 5′ nts are shown in bold, and 3′ nts in bold italics.

### D. Nucleotide composition of rRFs relates to their origin and putative binding modes

We selected the most prominent rRF length for 18S and 28S rRNA, 20nt (Supplementary Figure S3), and analyzed patterns of their binding with protein coding targets *ab initio* (45), using RNAhybrid (58). We encoded each binding nucleotide in an rRF as 1 (not binding as 0) and applied k-means clustering to obtain five distinct clusters (Figure 4 and Supplementary Figure S4). These clusters revealed major hybridization patterns that may be related to the mechanism of action of rRFs. While putative, such interactions have energy parameters close to miRNA-target binding, and they are supported by motifs found among most targets of each rRF (section E). Both 18S and 28S rRFs showed trends comparable to miRNAs (44) or tRFs (45), with interactions on either end of the guide RNA (and in some clusters close to the center or on both ends, Figure 4A, Supplementary Figure S4). Some rRFs had only the 5′ nucleotides interacting with targets, like the canonical miRNA seeds, although often being longer (up to 12nt, Figure 4 and Supplementary Figures S4, S6D). Non-canonical binding was observed on the 3′ end or in the middle of rRFs, and similar instances have also been reported for miRNAs (44,66,70) and tRFs (12,45,71). The strongest interactions involved nucleotides on both the 5′ and 3′ ends of the rRFs. Unlike siRNAs or plant miRNAs (pairing with full-length complementarity to targets), such rRFs hybridized near both ends with much weaker bindings in the middle.

We plotted cluster sequence logos to link their hybridization patterns with the nucleotide composition of the interacting sequences, revealing interesting compositional patterns. Positions of frequent binding had the highest information content (Figure 4B) and were mostly occupied by G (guanosine), followed by C (cytidine). A bias of the 5′ nt to adenosine and the 3′ site to pyrimidines was detected in both 18S and 28S rRFs. Several abundant rRF isoforms had many different targets and hence were overrepresented in the clusters. The exclusion of these top isoforms (Figure 4C) did not change the overall clustering and logo pattern, but numerous targets of these isoforms showed frequent lack of hybridization (possible gaps in rRF binding) between nt 9 and 12 (Figure 4C). A recent structure of Ago2-miRNA interaction (72,73) has suggested a similar binding mode, with a 5′ seed followed by an unpaired gap in miRNA positions 9–12 and an interaction with nucleotides in ‘supplementary chamber’ of Ago2. The GC-rich 3′ supplementary sites of those miRNAs may provide stronger binding to targets. rRFs were G-rich in the 5′ and 3′ binding sites but often had As or Us near the central gaps (Figure 4C) and more T→C conversions in potential Ago-crosslinking sites in PAR-CLIP (section E), compatible with the structural features of Ago binding to sRNA.

We compared the nucleotide distributions for rRF, tRF and miRNA guides forming CLASH hybrids with mRNAs, for mature rRNAs, and for guide-target paired bases, observing interesting similarities and differences (Table 3). All guides were also depleted for As in the paired regions. The paired rRF bases were predominantly Gs, with some contribution, especially in 28S rRFs, from Cs (Table 3A). This pattern resembled miRNAs, which also utilized Us for pairing (Table 3C). tRFs paired with targets mostly using Cs and some Gs (Table 3C). All unpaired rRF nucleotides were more evenly distributed, different from the overall rRNA composition.

The nucleotide content in flanking regions of rRFs may relate to their production, be that a directed biogenesis or a random decay. Similar to Figure 4 and Supplementary Figure S4, a typical cut site was seen between a pyrimidine and A (Table 3D). Pyrimidines accounted for 75% at the last nt at 3′ end of rRFs and 70% upstream of the 5′ end of rRFs. In both cases, the downstream nt was strongly biased to A (38% as the first base or 42% downstream base), hinting at an endoribonuclease involvement in the rRF production. Angiogenin (ANG), an endoribonuclease of the RNase A family, known to cleave rRNA into fragments of 100–500nt (38) is responsible for the biogenesis of tRNA halves under stress (29). ANG is a multifaceted protein, it can be excreted from and internalized by cells, it can shuttle between the cytoplasm and nucleus, where it binds rDNA intergenic spacers (74).

ANG (and similar ribonucleases) often cleave RNA between pyrimidine and adenosine, leaving the 2′,3′-cyclic phosphate (cp) at the 3′ end. A study of cp-containing RNA in stressed human cells has identified (in addition to tRNA halves) cprRFs frequently cleaved between pyrimidines (U>C) and adenosine (39). To be efficiently ligated to a 3′ adaptor (or their target in CLASH) rRFs should not contain 3′-cp. We compared the borders of CLASH rRFs with rRFs containing 2′,3′-cyclic phosphate (cprRFs), observed under stress conditions (39). Like rRFs (Figure 2, Table 3), cprRFs avoided large ES regions of rRNAs and their borders were mostly in the SS regions, also cut between C/U and A. The majority of CLASH rRFs had a counterpart among cprRFs (which were often longer but also had a high peak at 20 nt, Supplementary Figure S5) with either the same start, or the same end, or both. Remarkably, all rRFs with binding motifs (so less likely to be random, as described in the next section) had at least some cprRF reads with at least one exactly matching border, thus hinting at a possible mechanism. rRNA may be first cleaved by endoribonucleases in SS regions with a further breakage or cleavage of such longer cprRF-like intermediates in SS or DS stretches, giving rise to rRFs with SS-DS ends and SS-only ends (Table 1). The cut site similarity between these different protocols further suggests that target-binding rRFs are often preserved in CLASH.

We note that RNase A/T1 digestion step in CLASH might affect the C/U preferences above, but this cannot explain the observed frequency of A on the other side of the cut. Furthermore, rRF, miRNA and tRF guides in CLASH showed preference to A and U (albeit with different frequencies) at 5′ (bold in Table 3D–E), but divergent nts on 3′-end (bold italics in Table 3D–E). This argues that observed ends are not a result of RNase A/T1 treatment but of some guide-specific biological processes. For example, the 5′ terminal nts of miRNA CLASH guides were mostly Us, consistent with structural studies (75) in human. Bias to U or A on the 5′ end of Ago-loaded guide has been shown for human miRNAs (75) and is also seen in our results for tRFs (Table 3), thus it may be a common, structure-driven, feature of different Ago-loaded sRNAs.

### E. rRF interaction regions contain specific binding motifs, compatible with rRF-Ago crosslinking patterns

As shown earlier, tRFs and their targets contain complementary motifs, which we predicted to act as target interaction sites (45). In the three most abundant CLASH tRFs the positions of seed regions have been identified experimentally (46) and all of these matched our predictions. We also have found such seed-matching predicted motif (45) in miR-1983, which is a tRF (straddling the border of IleTAT tRNA) loaded to Ago (47). Encouraged by such validation, we applied this predictive approach for searching motifs in targets of rRFs (Figure 1A).

Motif search was independent of the binding patterns above (Figure 4) and served as a check for non-randomness of rRF-target pairs. For each rRFs isoform we compiled its target list using chimera-contained fragments of all its target genes. In each list we searched for common motifs not taking into account the rRF sequence. The motifs enriched in targets were then matched back to the isoform sequence using FIMO (63), as another independent test of the rRF-target binding (*P*-value cutoff 0.001), typically consistent with the RNAhybrid prediction of hybridization. This procedure and the fact that almost all targets contained such a motif strongly indicated that the corresponding rRF-target interactions were very likely. Further, more than a single occurrence of the same target gene (with variably cut subsequences of the same gene across chimeras yet containing the same motif) was detected for many rRFs. Multiple independent occurrences of subsequences of the same target with the same rRFs pointed to biologically reproducible interactions, not PCR artifacts.

In total, we found 680 motifs significantly enriched in such target lists, matching corresponding rRFs. These motifs were supported by 27 340 unique rRF-target pairs and 108,772 total reads, indicating likely binding. Not all rRF-target pairs produced a motif: rRFs either had <5 targets, or their target lists did not reveal significant common motifs, or such common motifs failed to match back to rRFs. These 680 motifs targeted 8085 unique genes (considering all chimeric reads). We also produced a high confidence set of 1382 target genes (with high read support and high unique chimera counts, as defined in Methods). 99.5% (1375/1382) of the high confidence targets had a motif, versus 73% for the rest of unique genes. However, an absent motif did not always indicate a lack of interactions: for example, 140 rRFs had >50 reads supporting a very likely interaction with a single target (and <5% total reads supporting 1–3 other targets, Supplementary Table S6).

To provide details of 680 motif-containing rRFs in a compact form, we binned them into 89 groups (each including one or a series of overlapping rRFs, up to 57 per group). Here, we clarify the nature of these groups on a few examples. Members of the same group usually had different end positions but often very similar motifs, for example, for the rRFs with the most of unique targets (Figure 4C, Supplementary Figure S6). The nts rarely present in motifs (smallest letters) matched the binding gap revealed by RNAhybrid (Figure 4C, Supplementary Figure S6). The 7-nt motif at the 5′ end of the rRF 18S-1446–1464 resembled a miRNA seed, although the RNAhybrid pattern (red line) showed a potential 3′ extended binding (Supplementary Figure S6D).

Some groups had a more complex arrangement of rRFs and motifs. The region in Supplementary Figure S6A is in fact a part of a group 18S-910–960 (Supplementary Figure S7A), illustrating how larger groups are merged. 29 rRF isoforms (93.5% of reads, including the most abundant 18S-919–937) are seen on the 5′ end, with most rRF starts in the interval 910–922 and ends in 933–941. All these rRFs supported a common motif at 924–934. Note that motifs for each rRF were generated independently and their significant overlap (Supplementary Figure S7A) is another striking indication of the non-randomness in the binding regions. A secondary 3′ motif was found in a single rRF 18S-938–960, which clustered in the same group due to 5% of the total group reads, including very long rRFs (e.g. 18S-919–954), joining the group members (Supplementary Figure S7A). 18S-938–960 had 0.8% of the group reads but that still equaled ∼100 reads supporting a significant motif (E-value = 2.8E–4, *P*-value = 1.6E–13). There is a possible analogy: functional miRNAs are primarily formed from one (‘mature’) strand of pre-miRNA and loaded to Ago, while the other strand (called ‘passenger’, or ‘star’) is often non-functional and degraded, although switches of those strands in Ago loading do occur, for example, with aging (76). Moreover, long pre-miRNAs have been suggested to directly associate with Ago (51,77,78).

We averaged nt frequencies across all motifs in a group, scaled the height of the logo letters by the number of motif-supporting reads for every position and showed a combined motif for every group (Supplementary Figure S8). Hence, secondary motifs (such as the 3′ motif of group 18S-910–960) are seen there as smaller logos. Several groups have secondary motifs (Supplementary Figure S8) and typical arrangements of rRFs in them are detailed in Supplementary Figure S7. Some of these arrangements are compatible with the miRNA analogy above, although the longer rRFs crossing several motifs may also be experimental artifacts or may reflect other complexities of the rRF-target interactions. For completeness, we presented all 680 motifs and containing rRFs in this compact and unbiased manner, ranked by different parameters (top 20 in each category shown in red font in Supplementary Figure S8). The group logos represent propensities of rRF nucleotides to be involved in targeting, derived from multiple independent detections of motifs. Each group did bind high confidence targets (averaging 79 per group; with many genes targeted by multiple rRFs this number is >1375/89 ≈ 15.5).

We also found motifs that mapped to multiple locations in the same rRF sequence, for example, a 5-nt motif CCTGG found near the 5′ end and in the middle of the group 18S-1–37 (underlined in Supplementary Figure S8), and both motifs overlapped with binding regions of RNAhybrid. We observed two other groups, ETS1–3654-3675 and ITS2–1167-1204, with repeated motifs. This resembled our findings for tRFs, with a short motif occurring twice in the sequence of LeuAAG tRF-3 (45).

**Figure 5.**
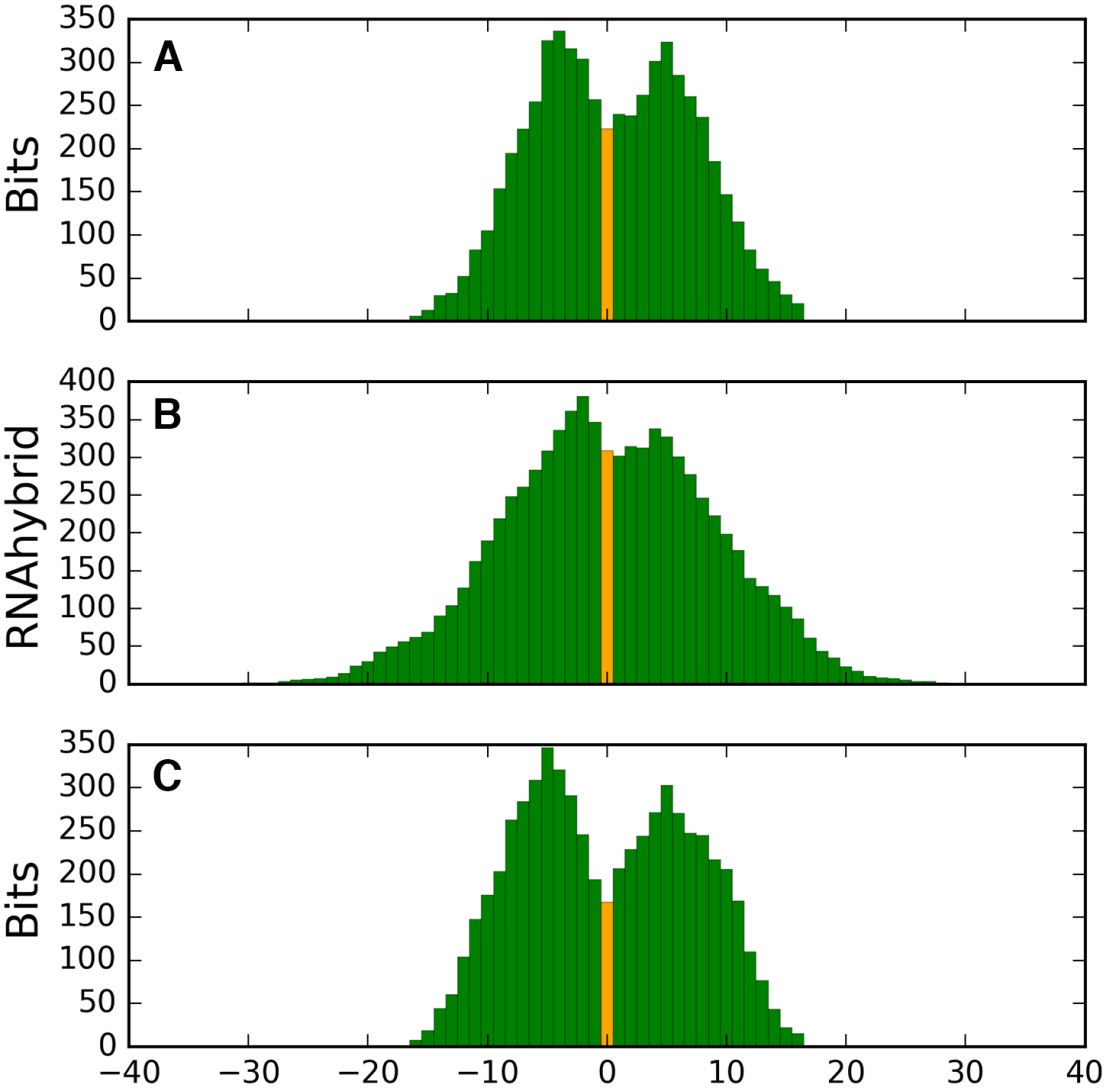
Positions of T→C conversion sites are compatible with the predicted interaction motifs and show conservation between species. (**A**) Cumulative bitscores and (**B**) hybridization frequencies relative to the conversion sites in human PAR-CLIP. (**C**) Cumulative bitscores in human motif positions relative to the conversion sites in mouse PAR-CLIP. Conversion sites positioned at zero and shown in light orange. See Supplementary Figure S8 for specific positions for each motif.

We analyzed available Ago1 to Ago4 PAR-CLIP datasets in HEK293 cells (49) and detected large numbers of rRFs there. 4-thyouridines-modified residues often change to cytidines crosslinking with RNA binding proteins in PAR-CLIP datasets, which have been used to detect target binding in miRNAs (49) and tRFs (14). Target-paired sRNA regions are expected to be depleted in (and adjacent to) Ago crosslinking sites and we compared patterns of T→C conversions to the identified motifs. A high conversion frequency could be seen in the rRF regions that were rarely paired to the targets (see black arrows in the examples in Supplementary Figure S6). To analyze this connection for all rRFs, we aligned all motifs placing the most frequent T→C conversion site at position 0 and plotting the cumulative information content from the motif for all rRFs (i.e. stacking the bitscores for each position, Figure 5A). The most frequent conversion site was typically some 3–6nt downstream or 5–7nt upstream of the most conserved motif positions. Analogously, when we used in the same alignments to graph the cumulative fraction of targets hybridizing to each position of motifs, we observed a similar plot shape, showing reduced target binding at the conversion site (Figure 5B).

### F. Mouse Ago2-IP and human CLASH samples contain rRFs originating from homologous rRNA regions, they are detected in the cytoplasm and nucleus of murine cells

**Figure 6.**
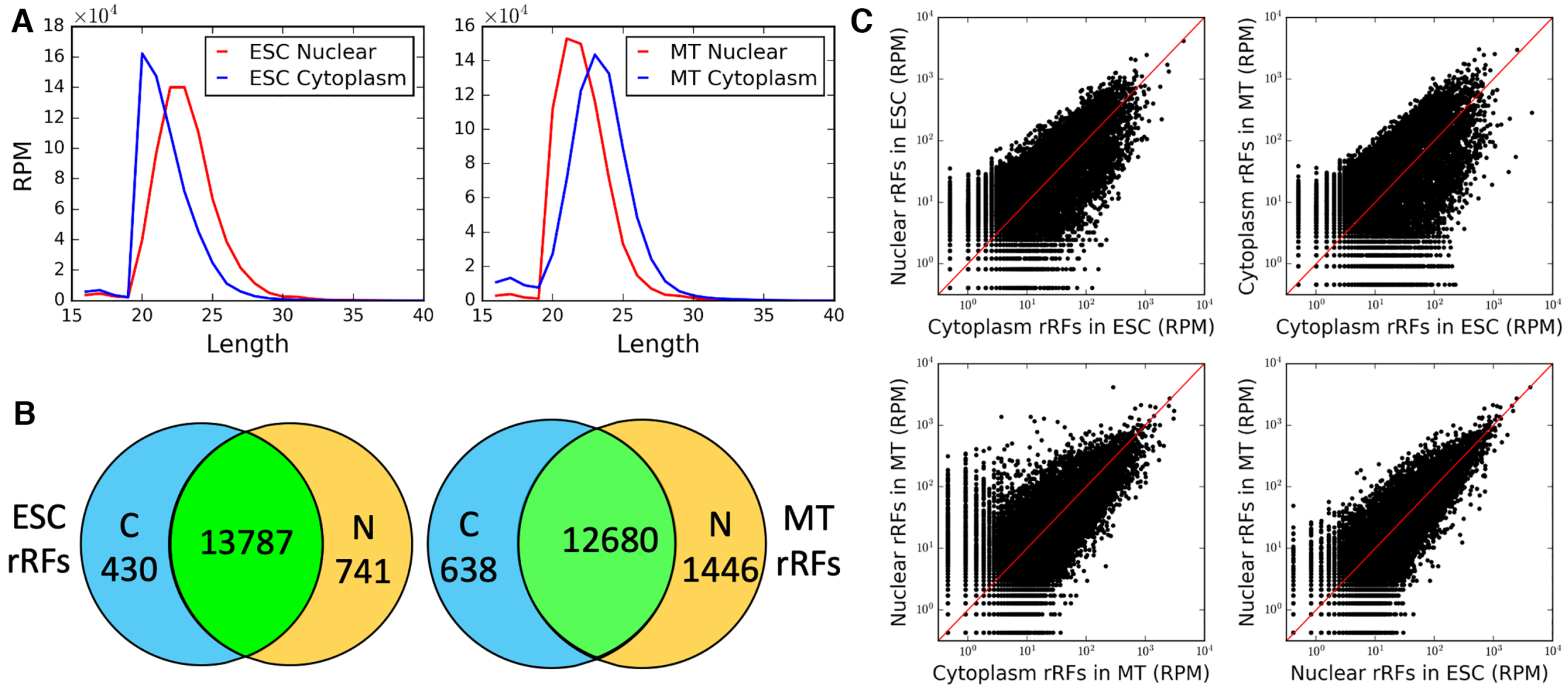
The same Ago-bound rRFs are found in both the cytoplasm and nucleus of mouse cells. (**A**) Length distributions of cytoplasmic (blue) and nuclear (red) rRFs. (**B**) Venn diagrams of rRFs at RPM≥5. (**C**) Profiles of rRF abundance comparing the cytoplasm and nuclear fractions (left) and the two murine cell lines (right).

We sought confirmations of our findings in other species. In mouse, we detected rRFs in Ago2 PAR-CLIP datasets of mESC and C2C12 myocytes (MT) (50). As in human rRFs (Figure 1E, Supplementary Figure S3), mouse rRF lengths peaked at 20 nt in cytoplasmic Ago2 in mESC cells. rRFs in cytoplasmic Ago2 in MT, nuclear Ago2 in mESC and MT showed peaks of 20–22nt (Figure 6A). Murine Ago2 reads mapped to the regions in mouse rRNAs, largely corresponding to the peaks of human Ago1 rRFs and covering the motif groups we identified in section E (Supplementary Figure S2). We note that in *Drosophila* many miRNAs load to both Ago1 and Ago2, with dominant isoforms often showing difference in length for the same miRNAs (76), and that similar overlaps have been reported for tRFs (16) and rRFs (42). So it was encouraging to see 37% of murine Ago2 rRFs overlapping motif-containing human Ago1 rRFs (Supplementary Figures S2, S8) and 99.7% overlapping at least one human rRF.

We made several important observations from the analyses of these murine datasets. First, an absent band on a gel with radiolabeled RNAs recovered from FLAG-IP suggested there were no sRNAs (and thus no rRFs) detected in either nucleus or cytoplasm with a loading-deficient Y529E Ago2 mutant, in contrast to a non-mutated Ago2 (see Figure 3D, F in (50)). This provided a negative control, arguing against a random, non-specific association of rRFs with Ago2 complexes in either cellular compartment. The Y529E Ago2 mutant localized only to the cytoplasm, while sRNA-loaded Ago2 was also able to enter the nucleus (50), and up to 20% of human Ago2 has been detected in the nucleus of HEK293 cells in the same study.

Second, we found >90% of mouse rRFs to be common and showing comparable profiles between the cytoplasmic and nuclear Ago2 PAR-CLIP samples (Figure 6B). Both nuclear and cytoplasmic samples showed similar patterns of intersection with the human motif groups (colored boxes in Supplementary Figure S2). This placed >50% of Ago-rRF complexes to the nucleus, away from translating ribosomes. It was also consistent with the intron targeting by rRFs described in section C and resembled Ago2-miRNA targeting shown for nuclear transcripts (50).

Third, when we mapped T→C conversions in mouse rRFs, they matched a majority of the same locations seen in CLASH rRFs (Supplementary Figure S8), displaying the same distribution of motif information content around them as in human (Figure 5C). This showed that Ago-rRF crosslinking in a different species and with a different Argonaute protein was consistent with the binding motifs we detected in CLASH. We combined into a single display the PAR-CLIP crosslinking in human and mouse with motifs and target hybridization sites (Supplementary Figure S8). We also observed a depletion of mouse rRFs in the mammalian rRNA expansion segments, similar to CLASH rRFs (Supplementary Figure S2). These clear cross-species parallels, together with the earlier reports on rRFs in eukaryotes (18–22), suggest a certain level of conservation of rRF-related mechanisms, which could be expected from such ancient molecules as rRNA, and hint at a potential role for rRFs in the other kingdoms of life.

All of these observations strongly supported the rRF findings in CLASH and provided important evidence of the cellular distribution of Ago-bound rRFs, compatible with their putative regulatory function. Such distribution between the cytoplasm and nucleus strongly argues against a random and non-specific formation of Ago-rRF complexes, as further discussed in section J.

### G. Characterization of protein-coding targets of rRFs

In CLASH hybrids, we observed various mRNAs targeted by rRFs in high numbers, with different target support for forward and reverse pairs (Table 2). The most abundant rRF-mRNA interactions involved ENOX1 gene and a translation initiation factor CTIF in reverse pairs (Supplementary Table S3). Histone genes comprised a prominent group of targets, with HIST2H2AA3 in the top ten most frequent targets in both orientations (with slightly more frequent HIST2H3A in reverse). 4.6% of all 166 653 unique rRFs-mRNA interactions involved different histone mRNAs, in 3′ UTR (68%) or CDS (29%) regions. Notably, ribosomal proteins (RPs), translation initiation factors and elongation factors were observed as targets of rRFs (see examples in Supplementary Table S3).

**Figure 7.**
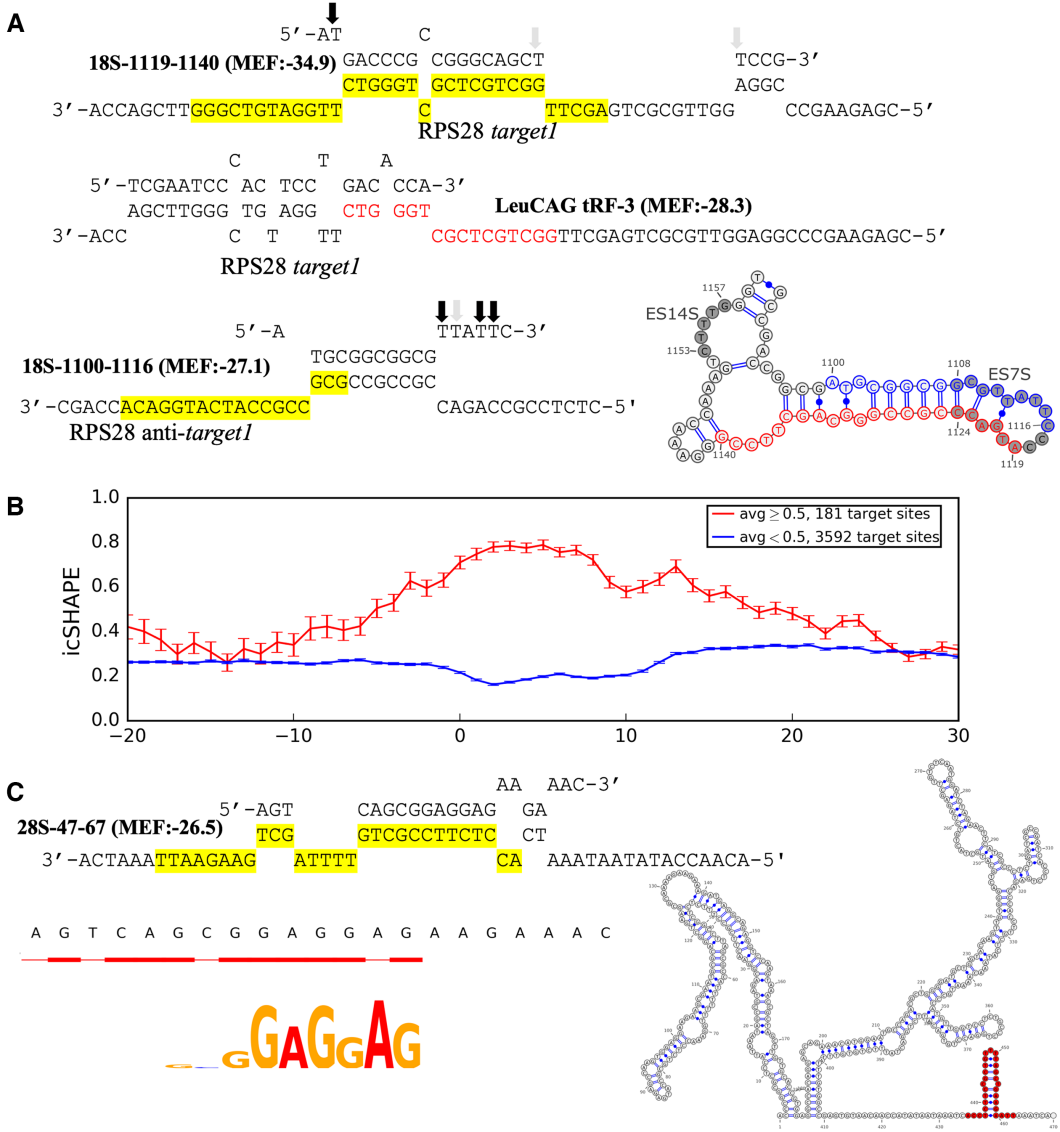
Secondary structure of putative rRFs-target interaction sites. (**A**) Secondary structure of the interactions predicted by RNAhybrid between 18S-1119–1140 (top) and 18S-1100–1116 (bottom) and the entire RPS28 transcript. The target sequences of the rRFs identified in chimeric reads are highlighted and the rRF match is shown in red on the tRF target (middle). Interaction between the tRF and RPS28 is shown as reported earlier (24). The arrows show the frequency (darker shade being more frequent) of T→C conversions in PAR-CLIP data. The secondary structure of ES7S hairpin and ES14S (shaded circles) is shown in the bottom right corner. The origins of the two complementary rRFs targeting the RPS28 transcript, are colored in blue and red. (**B**) Secondary structure of rRFs target sites. Only the motif-containing target sequences identified in the unique rRF-target pairs are included. Relative position to the motif is revealed on the x-axis where 0 represents the start position of the motif in target sequences. Averaged icSHAPE score (0 indicates double-strandedness and 1 indicates single-strandedness) across all targets is shown on the y-axis, with SEM error bars. Targets are separated into two groups based on these icSHAPE scores averaged over the motif region: <0.5 (blue line) or not (red line), and each group shows an increased propensity to adopt its corresponding structure in the motif region. (**C**) The best (lowest MFE) secondary structure of the interaction between 28S-47–67 and the entire RPL41 mRNA. Motif and hybridization pattern revealed by multiple rRF-target chimeras are shown in the bottom left corner. CLASH target (highlighted, top) is shown in red in the secondary structure of RPL41 (right).

One of the RP targets, RPS28, has been reported to be regulated by tRF LeuCAG with an interesting mode of action, which involves binding to and unfolding the secondary structure and thus enhancing translation of the RPS28 transcript (34). Strikingly, we found that rRF 18S-1119–1140 may form an even stronger interaction with one of the described target sites of that tRF, *target1*, see Figure 5A in (34). The tRF binding site in the RPS28 transcript predicted by RNAhybrid overlapped by 6 bases (CTGGGT) with the target sequence identified in CLASH chimeric reads (tail of tRF match was the start of the rRF match, see Figure 7A). Interestingly, we found another rRF isoform 18S-1100–1116 (part of ES7S, Figure 7A) interacting with the anti-*target1* (translation initiation site) of the RPS28 mRNA. The two adjacent rRFs were cleaved from a small hairpin structure of the 18S rRNA and thus had a partial complementarity in their sequences, which may be responsible for their interaction with the duplex structure of the mRNA. Both rRFs had too few distinct target genes to infer a binding motif, however, we observed the highest T→C conversion rates were at the 5′ end of 18S-1119–1140 and the 3′ end of the 18S-1100–1116, excluded from the target binding regions. All of this suggests that rRFs might be also involve in regulating RPS28 translation by a mechanism comparable or related to that of LeuCAG tRF (also see section J).

We reasoned that rRFs may interact with other transcripts in a similar fashion, hence we analyzed the intra-molecular secondary structures of rRF targets using data of *in vivo* click selective 2′-hydroxyl acylation and profiling experiments (icSHAPE) for the cytoplasm of HEK293 cells (48). We aligned all target genes with motifs such that the motif starts were at the same position and used it as a zero coordinate on the x-axis. We then plotted the average icSHAPE score for each position of the target genes (Figure 7B). We separated these genes into two groups: targeted by rRFs in double-stranded (*s*<0.5, blue line in Figure 7B) and in SS regions (*s* ≥ 0.5, red line). Strikingly, we found that both lines went to their extremes over the motif length compared to the flanking regions, suggesting that interactions occur in regions with well-defined structural changes. Such secondary structures showed a clear bias as almost 95% of target motif sites with available icSHAPE data (3592/3773) were in DS regions. To confirm that this effect was not an artifact of our separation procedure, we compared the motif set to a set of random 12-mers generated from rRF target genes, similarly split by their icSHAPE score. The target DS motifs showed significantly (*P*-value < 1E–16) higher drop in icSHAPE relative to their up- and downstream regions, compared to that in random DS 12-mers (mean drop of 0.07 over the motif region but up to 0.2 at some nucleotide positions in the motif versus an invariable mean drop of 0.01 in the random set). The icSHAPE score for SS motifs relative to their surroundings was only slightly higher than random, was not significant (*P*-value = 0.5) and accounted for just 5% of motifs.

These structural differences may potentially reflect biases in icSHAPE score sampling or be due to a genuine preference by some rRFs for targeting DS mRNA regions. In addition to the non-Ago action of the LeuCAG tRF above, we note a report of Ago-loaded miRNAs interacting with their target sites after a translating ribosome unfolds them (79). Among the putative rRF DS targets, we noted an interesting case of targeted duplex structures in mRNAs of multiple ribosomal proteins (RPL41 >> RPL39 > RPL4) of a large subunit. E.g., RPL41 was targeted by 28S-47–67 in a small helix in the 3′ UTR (Figure 7C). Other rRFs from the same group (like 28S-51–67) had similar motifs matching the longest targeted segment, which had the lowest interaction MFE of the entire mRNA sequence and such rRFs. Targeting of such small helices may be representative of the general trend of DS targeting (Figure 7B). On the other hand, 5% of targets showed an increase in single-strandedness in the binding sites, suggesting there might be more than one mechanism of interaction with (and regulation by) rRFs. Such results also suggest that guide-driven regulatory processes may go beyond the traditional view of translational repression. For example, the latter cannot explain rRF-loaded Ago complexes in the nucleus, which we add to other reports of nuclear Ago (50,80–82). The motifs we identified and their target genes point to clear avenues for experimental validation and testing of molecular mechanisms of rRFs in future studies.

A regulatory role in the ribosome biogenesis has been reported for a human ATPase hCINAP. It may transiently bind to RPS14 protein to inhibit its association with the 18S pre-rRNA and to release the RPS14 to the properly rearranged pre-rRNA, when hCINAP is used to hydrolyze the ATP. The energy produced in the hydrolysis may stimulate the endonuclease activity of Nob1 to mediate the 18S rRNA maturation (83). We found that 18S-1600–1619 (Supplementary Figure S6B), in turn, may target the CDS of RPS14 mRNA and thus affect its translation, forming a potential feedback loop. Therefore, the post-transcriptional regulation of ribosome biogenesis may result from changes in both rRNA (destroyed by the fragmentation process) and encoded proteins (regulated by the fragments) and this may be a conserved process existing in plants (21). We found other components of the translation machinery, including initiation (CTIF, EIF1AX, EIF2S3, etc.) and elongation factors (EEF1B2 and EEF2), as putative targets of rRFs. It is possible that rRFs have a role in regulating the global translation activity and coordinating the cellular growth or malignant proliferation. Similar functions have been proposed for fly tRFs (35).

Regulatory effects of rRFs may be far-reaching, given that rRNAs constitute 80–90% of total cellular RNAs. To characterize the protein coding genes targeted by rRFs in CLASH in different contexts we performed Gene Ontology and KEGG pathway analysis. It showed significant enrichment of targets involved in various fundamental processes and pathways (Supplementary Table S7 for high confidence set, but trends remain for all genes), highlighting the breadth and importance of regulatory roles of rRFs. As expected from our observations above, ribosome biogenesis was among the significantly enriched terms and targets for both small and large subunits. Targets were enriched in such key complexes and pathways as spliceosome, proteosome, cell cycle, DNA replication, repair and multiple diseases related to these. Nervous system processes including neurogenesis, neuron differentiation and death, brain development and aging process also showed GO term enrichment, consistent with our earlier findings for age-associated rRFs in fruit flies (42).

### H. Certain rRFs abundant in cellular sRNA datasets are depleted in Ago-IP datasets

In addition to the datasets above, we analyzed eight additional cohorts of small RNA sequencing datasets or Ago-IP datasets in HEK293, HeLa and mouse cells (84–88). We detected abundant rRFs in the whole-cell and the nucleus sample of HeLa cells, suggesting that rRFs may be functional in different cell compartments. We observed no significant between-cohort correlation of the abundance of rRF isoforms in these datasets but frequent within-cohort correlations, likely pointing to differences in the experimental design or preparation/sequencing of the samples in individual labs (89). However, several specific fragments stood out in their patterns of presence in different rRF sets, showing effects independent of the factors above.

The most obvious difference was an overrepresentation of rRFs at the termini of mature rRNAs (Supplementary Table S8, Supplementary Figure S9), and this has also been reported in hundreds of human lymphoblastoid cell lines (40) and in other species (21,22,36,90,91). We observed considerable counts of rRFs at the 5′ end of the 28S rRNAs in all whole cell samples of HEK293 cells and HeLa. The relative abundance of such 5′ terminal 28S rRFs significantly dropped in the Ago datasets (Ago-IP, PAR-CLIP and CLASH, Supplementary Table S8), with a similar effect seen on a smaller scale on the 3′ ends of 28S and 18S (Supplementary Figure S9). The 5′ terminal 18S rRF showed a somewhat mixed pattern, with lower abundance in many Ago-IP but higher in PAR-CLIP samples, which led to an overall peak in Supplementary Figure S9. Another prominent difference was a rRF at the 01/A’ site, one of the endonucleolytic cleavage sites in the ETS1 region (92). This rRF was present in all non-Ago samples including cytoplasm, nucleus and Dicer/Drosha knockdown cells. A singular example of an rRF border possibly linked to the endoribonuclease activity during the rRNA maturation process, this rRF showed much lower levels or was absent in Ago-IP (Supplementary Table S8). As in human, such overexpression of the ETS1 rRF and 5′ terminal 28S rRFs was seen in the mESC whole cell sRNA (93) but not Ago2-IP (Supplementary Table S8). Both Ago and non-Ago datasets contained rRFs from the ends of 5.8S, with a large excess in non-Ago samples (Supplementary Figure S9), and a similar situation was seen in the 5S (not shown).

All these observations suggest that some rRFs are depleted in Ago-IP samples (such as those on the 5′ ends of mature rRNAs), despite being abundant among the cellular sRNAs. It remains to be seen if they are generated by a different mechanism, possibly related to rRNA maturation, given their excess on all rRNA ends and in the ETS1 region. However, their effects may still be related to the rRFs we described in the previous sections, as overexpressing in HeLa cells a 5′ rRF from the 28S rRNA inhibited several RPs, despite that rRF not being detected in Ago1-IP (41). It also suggests that sequencing only the total cellular sRNA or only the Ago-IP fraction may not provide an unbiased picture of a functional interactome of small RNAs originating from rRNAs (and likely from other abundant parental RNAs such as tRNAs).

### J. Further considerations: rRF generation, potential RNAi context, alternative hypotheses

To our knowledge, this is the first attempt to systematically characterize rRF targets and patterns of their binding using bioinformatics integration of multiple independent experimental datasets.

A regulation potential of rRNA has been noted before, with publications in 1990s reporting many hundreds of mRNAs hybridizing with numerous complementary subsequences in rRNAs in both human and mouse (94,95), leading to a development of the ribosome filter hypothesis (96). These papers have been published before the acceptance of RNAi and hence mostly discussed a potential binding between rRNA within a ribosome and mRNA during translation, with possible regulatory implications. We cannot exclude that some of the interactions we observed were a result of such processes. Not aiming to refute that hypothesis, we note that (i) many of the ‘ribosome filter’ interactions occurred in the expansion segments (94,95) and (ii) rRNA sequences localized to different parts of the ribosome would have different accessibility and spatial constraints on their interactions with mRNA in that model. Further, these interactions would not be seen in the nucleus. Hence our finding of rRFs in Ago1- and Ago2-IP in human and mouse cells and their pairing with thousands of targets puts these early observations in a different regulatory context, whereby an effect of any rRF would only be constrained by its abundance and sequence matches, as is the case with miRNAs and tRFs.

Despite all the evidence we presented above for rRFs being potential regulators, one should also consider a scenario, whereby rRFs may be captured in the Ago-IP pulldowns by accident. Targeted miRNA sites in mRNA have been shown to interact with miRNA-RISC after a translating ribosome unfolds them (79). This may place Ago directly on a collision course with ribosomes, which might result in damage to the latter, and a degradation of rRNA. However, such collisions would not be expected in the nucleus, where we observed Ago2-IP to contain the same rRFs in amounts comparable to the cytoplasm (Figure 6). Hence, the scenario of accidental pulldowns requires either (i) a massive influx of Ago complexes from the collision sites together with non-specific rRFs produced by collisions to the nucleus or (ii) a nuclear rRNA degradation producing rRFs identical to case (i), non-specifically sampled by the nuclear Ago-IP. Neither seems likely.

At every step in sections A–H, we tested for and found significant indicators of non-randomness among the properties of CLASH rRFs, resembling the patterns found in miRNAs or tRFs (44,45). This argues against rRFs being simple byproducts of mature rRNA biogenesis or random rRNA degradation. However, the process of rRF production remains unclear, is it a (i) ‘purposeful’ generation or a (ii) non-random decay, ‘organized’ to some extent? Let us first consider the option (i) and speculate on a possible biological pathway, starting with the best-known sRNA pathways.

The miRNA biogenesis requires two RNase III enzymes, Drosha and Dicer, to process primary miRNA to mature miRNA (97,98). tRFs have been suggested to be produced independently of Drosha and Dicer in multiple species (14,46,90,99), except for some Dicer1-dependent tRF-3s in human cell lines (8,10,11). Mutations in Dicer-2 and r2d2 (key components of siRNA pathway) may decrease the amount of some tRF types (14), and the ratio of short (20–22nt) to longer tRFs (35) in *Drosophila*. The loss of TRAMP-mediated RNA degradation leads to what has been called an ‘inappropriate entry’ of rRFs and tRFs into the RNAi pathway in fission yeast (100). Since then, tRFs have been accepted as post-transcriptional regulators, detected in the cytoplasm and nucleus (14,101), in Ago or not (14,46,101), and found targeting introns in CLASH (45). Similarly, the data we presented here argue for rRFs being legitimate entrants into RNAi. Drosha, Dicer and Ago have been detected in the nucleus and implicated in processing pre-rRNA to mature rRNA (102,103). We report here our findings of (i) Ago2-loaded rRFs being abundant in the nucleus of two murine cell lines, (ii) introns pairing with rRFs in CLASH and (iii) elevated rRF counts in the HeLa nucleus. Hence, it is possible that Drosha/Dicer/Ago are involved in the rRFs processing and that rRFs participate in post-transcriptional regulation in the cytoplasm and nucleus.

Dicer is dispensable for generating the mouse terminal rRFs and rRFs bound to P19 protein (37). A singular ETS1 rRF showed increased abundance in shDicer and noDicer HEK293 samples and in the HeLa nucleus sample (Supplementary Table S8), although it was not found in CLASH. Given that the pre-5.8S rRNAs accumulated but the mature 5.8S rRNA level remained unchanged in HeLa cells depleted of Drosha/Dicer, the latter have been speculated to be responsible for degrading excessive precursors (103). It is conceivable that such degradation may be ‘purposeful’, and the processing of some precursor rRNAs by Drosha/Dicer results in forming rRFs instead of mature rRNAs.

Ago proteins are best known for their sRNA loading to form RNA-induced silencing complex (RISC). The original CLASH publication has focused on miRNA-target interactions, detected as chimeric pairs, confirming known cases but also finding non-canonical patterns of binding (44). Detecting tRFs loaded to Ago in different species has served as one of the indications of their functional role (14,16), and multiple parallels have been drawn with miRNAs. Earlier, we have identified motifs significantly enriched among tRF targets in the CLASH dataset, with similarities to miRNA seed regions (45). Here, our findings of rRFs in CLASH and mouse Ago2-IP suggest that rRFs are also loaded to Ago, and thus may function as guide RNAs in RISC. Ago crosslinking sites in human and mouse often flanked the motifs (Figure 5, Supplementary Figures S6, S8), providing further support to these likely target-binding sites of rRFs. However, binding motifs require experimental validation. By themselves, they do not indicate the same function but raise intriguing questions. Do miRNAs, tRFs and rRFs act differently on their targets? Do sRNAs compete for, or control each other's Ago loading? Age-dependent patterns of Ago loading of miRNAs, tRFs and rRFs in *Drosophila* (16,42,76) or a balance between piRNA and 22-nt rRFs seen in the nematode (104) suggest such a possibility. Are agotrons targets of sRNA or longer regulatory (and possibly competing) RNAs?

Given the large number of rRFs, their modes of action may vary. In mouse P19 IP, rRFs are enriched but unrelated to the canonical siRNA-like post-transcriptional gene silencing (37). Given the diverse rRFs profiles in distinct cellular components and differences with the Ago-IP datasets (Supplementary Table S8), we expect the functionality of rRFs to be sequence-, tissue- and species-specific and dependent on the effector protein that they associate with (be that Ago or others, like P19). There is also a possibility that some CLASH pairings reflect interactions not mediated by Ago. A targeting model for RPS28 we described (Figure 7A) has two adjacent rRFs homing in on a *target1* sequence described as binding LeuCAG tRF (34). This tRF has been reported to act differently than a miRNA, not loading to Ago. Predicted rRF binding to *target1* is even stronger, thus it might well compete with the tRF in the same (unspecified) manner, without Ago involvement. However, even if some rRF-target interactions reported here are not mediated by Ago, the respective motifs we present should still be relevant for further experimental studies of rRF-target binding.

Finally, both 5S rDNA and 45S rDNA are tandemly repeated on chromosomes and their copy numbers (CN) vary significantly across species (or even across human genomes). A remarkably concerted copy number variation (cCNV) between 5S and 45S rDNA reported in normal tissues in human and mice has led to the interpretation that there is a mechanism maintaining balanced levels of rRNAs due to stoichiometric constraints (105). Such tightly coupled rDNA dosage in genome is disrupted in diseases, for example, in different types of carcinomas the 5S rDNA CN goes up while the 45S rDNA CN decreases (106). The line of reasoning linking these effects with disease has been focused on ribosomal stress (106) although the increased proliferation in tumors is not obviously connected to a disbalance of rRNA stoichiometry. We speculate that such deviations of cCNV might also lead to regulatory changes in disease due to the disruption of the levels or rRFs. Consistent with this idea, published analyses of whole genomes of other species have reported a fixed derived deletion affecting only a part of 5S rDNA in woolly mammoths (107) and a partial 5S duplication in a large proportion of cattle breeds (108).

In conclusion, the emerging field of study of regulatory fragments originating from well-known molecules with ‘textbook’ functionality shows a surprising complexity in terms of the source of such fragments (multiple specific locations on tRNA and rRNA molecules), target types and potential mechanisms of regulation.

## Supplementary Material

gkab190_Supplemental_Files
