# Supplementary material for: Computational meta-analysis of ribosomal RNA fragments: potential targets and interaction mechanisms
Source: Nucleic Acids Res. 2021 Mar 27;49(7):4085–103. doi: 10.1093/nar/gkab190 (PMC8053083; doi:10.1093/nar/gkab190)
Supplement: gkab190_Supplemental_Files [file gkab190_supplemental_files.zip › FigureS8.pdf]

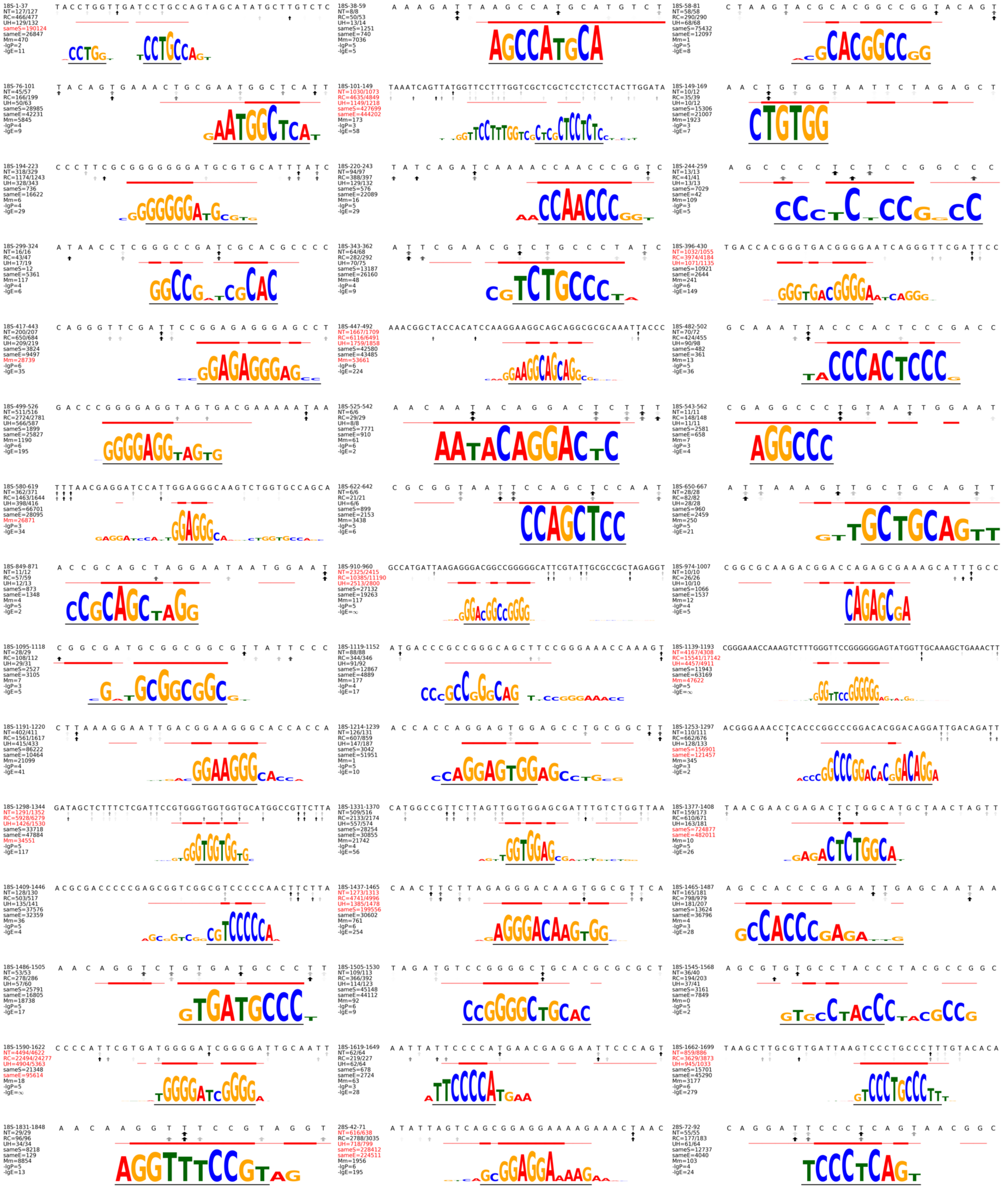

18S-1-37 NT=127/127 RC=466/477 UH=129/132 sameS=190124 sameE=26847 Mm=470 -lgP=2 -lgE=11
18S-38-59 NT=8/8 RC=50/53 UH=13/14 sameS=1251 sameE=740 Mm=7036 -lgP=5 -lgE=5
18S-58-81 NT=58/58 RC=290/290 UH=68/68 sameS=75432 sameE=12097 Mm=1 -lgP=5 -lgE=8
18S-76-101 NT=45/57 RC=166/199 UH=50/63 sameS=28985 sameE=42231 Mm=5845 -lgP=4 -lgE=9
18S-101-149 NT=1030/1073 RC=4635/4849 UH=1149/1218 sameS=427699 sameE=444202 Mm=173 -lgP=3 -lgE=58
18S-149-169 NT=10/12 RC=35/39 UH=10/12 sameS=15306 sameE=21007 Mm=1923 -lgP=3 -lgE=7
18S-194-223 NT=318/329 RC=1174/1243 UH=328/343 sameS=736 sameE=16622 Mm=6 -lgP=4 -lgE=29
18S-220-243 NT=94/97 RC=388/397 UH=129/132 sameS=576 sameE=22089 Mm=16 -lgP=5 -lgE=29
18S-244-259 NT=13/13 RC=41/41 UH=13/13 sameS=7029 sameE=42 Mm=109 -lgP=3 -lgE=5
18S-299-324 NT=16/16 RC=43/47 UH=17/19 sameS=12 sameE=5361 Mm=117 -lgP=4 -lgE=6
18S-343-362 NT=64/68 RC=282/292 UH=70/75 sameS=13187 sameE=26160 Mm=48 -lgP=4 -lgE=9
18S-396-430 NT=1032/1055 RC=3974/4184 UH=1071/1135 sameS=10921 sameE=2644 Mm=241 -lgP=6 -lgE=149
18S-417-443 NT=200/207 RC=650/684 UH=209/219 sameS=3824 sameE=9497 Mm=28739 -lgP=6 -lgE=35
18S-447-492 NT=1667/1709 RC=6116/6491 UH=1759/1858 sameS=42580 sameE=43485 Mm=53661 -lgP=6 -lgE=224
18S-482-502 NT=70/72 RC=424/455 UH=90/98 sameS=482 sameE=361 Mm=13 -lgP=5 -lgE=36
18S-499-526 NT=511/516 RC=2724/2781 UH=566/587 sameS=1899 sameE=25827 Mm=1190 -lgP=6 -lgE=195
18S-525-542 NT=6/6 RC=29/29 UH=8/8 sameS=7771 sameE=910 Mm=61 -lgP=6 -lgE=2
18S-543-562 NT=11/11 RC=148/148 UH=11/11 sameS=2581 sameE=658 Mm=7 -lgP=3 -lgE=4
18S-580-619 NT=362/371 RC=1463/1644 UH=398/416 sameS=66701 sameE=28095 Mm=26871 -lgP=3 -lgE=34
18S-622-642 NT=6/6 RC=21/21 UH=6/6 sameS=899 sameE=2153 Mm=3438 -lgP=5 -lgE=6
18S-650-667 NT=28/28 RC=82/82 UH=28/28 sameS=960 sameE=2459 Mm=250 -lgP=5 -lgE=21
18S-849-871 NT=11/12 RC=57/59 UH=12/13 sameS=873 sameE=1348 Mm=4 -lgP=5 -lgE=2
18S-910-960 NT=2325/2415 RC=10385/11190 UH=2513/2800 sameS=27132 sameE=19263 Mm=117 -lgP=5 -lgE=∞
18S-974-1007 NT=10/10 RC=26/26 UH=10/10 sameS=1066 sameE=1537 Mm=12 -lgP=4 -lgE=5
18S-1095-1118 NT=28/29 RC=108/112 UH=29/31 sameS=2527 sameE=3105 Mm=7 -lgP=3 -lgE=5
18S-1119-1152 NT=88/88 RC=344/346 UH=91/92 sameS=12867 sameE=4889 Mm=177 -lgP=4 -lgE=17
18S-1139-1193 NT=4174/4308 RC=15541/17142 UH=4457/4911 sameS=11943 sameE=63169 Mm=47622 -lgP=5 -lgE=∞
18S-1191-1220 NT=402/411 RC=1561/1617 UH=415/433 sameS=86222 sameE=10464 Mm=21099 -lgP=4 -lgE=41
18S-1214-1239 NT=126/131 RC=607/859 UH=147/187 sameS=3042 sameE=51951 Mm=1 -lgP=5 -lgE=10
18S-1253-1297 NT=159/173 RC=662/676 UH=128/133 sameS=156901 sameE=121457 Mm=345 -lgP=3 -lgE=2
18S-1298-1344 NT=1291/1352 RC=5928/6279 UH=1426/1530 sameS=33718 sameE=47884 Mm=34551 -lgP=5 -lgE=117
18S-1331-1370 NT=509/516 RC=2133/2174 UH=557/574 sameS=28254 sameE=30855 Mm=21742 -lgP=4 -lgE=56
18S-1377-1408 NT=159/173 RC=610/671 UH=163/181 sameS=724877 sameE=482011 Mm=10 -lgP=5 -lgE=26
18S-1409-1446 NT=128/130 RC=503/517 UH=135/141 sameS=37576 sameE=32359 Mm=36 -lgP=5 -lgE=4
18S-1437-1465 NT=1273/1313 RC=4741/4996 UH=1385/1478 sameS=199556 sameE=30602 Mm=761 -lgP=6 -lgE=254
18S-1465-1487 NT=165/181 RC=798/979 UH=181/207 sameS=13624 sameE=36796 Mm=4 -lgP=3 -lgE=28
18S-1486-1505 NT=53/53 RC=278/286 UH=57/60 sameS=25791 sameE=16805 Mm=18738 -lgP=5 -lgE=17
18S-1505-1530 NT=109/113 RC=366/392 UH=114/123 sameS=45148 sameE=44112 Mm=92 -lgP=6 -lgE=9
18S-1545-1568 NT=36/40 RC=194/203 UH=37/41 sameS=3161 sameE=7849 Mm=0 -lgP=5 -lgE=2
18S-1590-1622 NT=4494/4622 RC=22494/24277 UH=4904/5363 sameS=21348 sameE=95614 Mm=18 -lgP=5 -lgE=∞
18S-1619-1649 NT=62/64 RC=219/227 UH=62/64 sameS=678 sameE=2724 Mm=63 -lgP=3 -lgE=28
18S-1662-1699 NT=859/886 RC=3629/3873 UH=945/1033 sameS=15701 sameE=45290 Mm=3177 -lgP=6 -lgE=279
18S-1831-1848 NT=29/29 RC=96/96 UH=34/34 sameS=8218 sameE=129 Mm=8854 -lgP=5 -lgE=13
28S-42-71 NT=616/638 RC=2788/3035 UH=718/799 sameS=228412 sameE=224511 Mm=1956 -lgP=6 -lgE=195
28S-72-92 NT=55/55 RC=177/183 UH=61/64 sameS=12737 sameE=4040 Mm=103 -lgP=4 -lgE=24

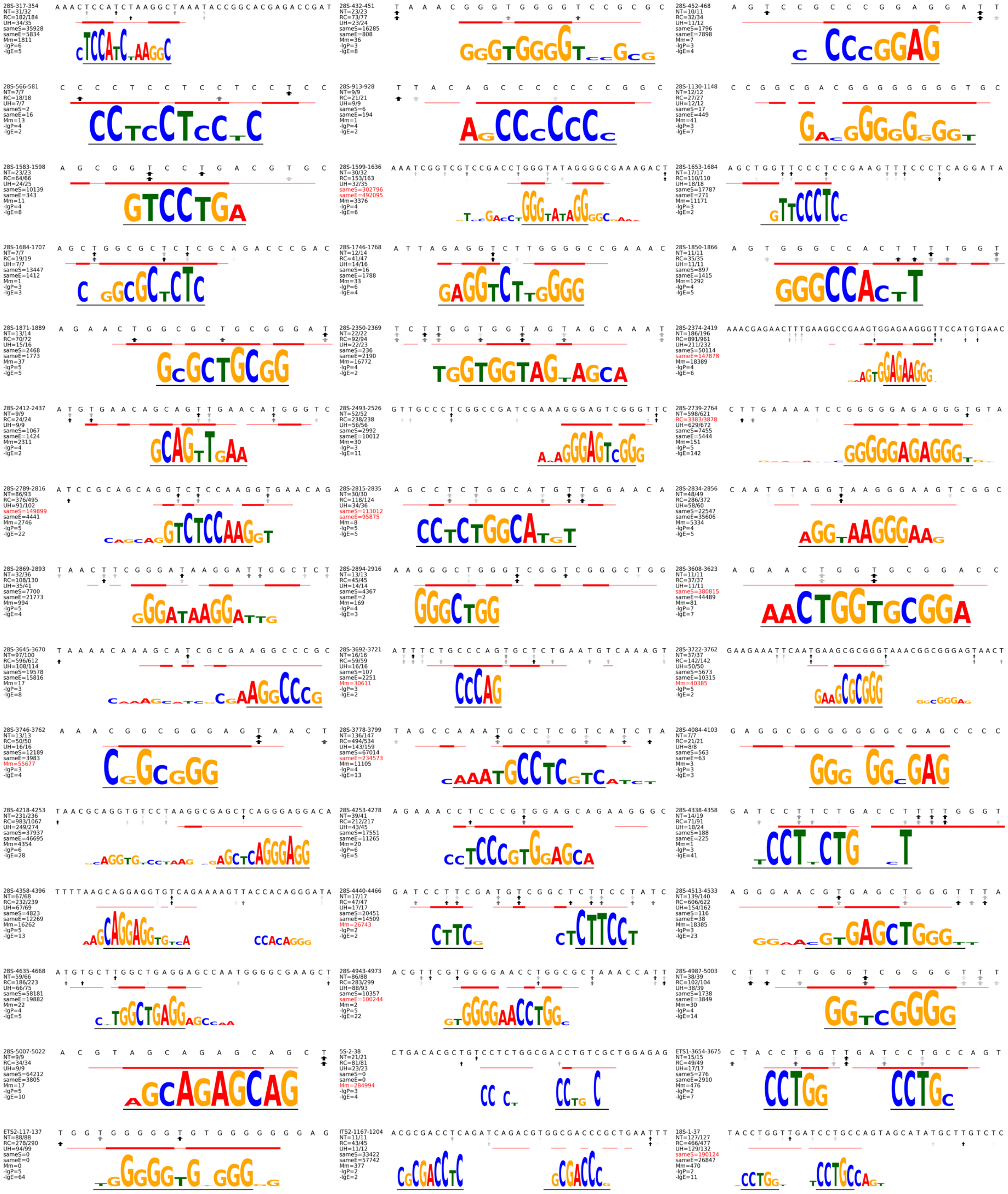
28S-317-354
28S-432-451
28S-452-468
28S-566-581
28S-913-928
28S-1130-1148
28S-1583-1598
28S-1599-1636
28S-1653-1684
28S-1684-1707
28S-1746-1768
28S-1850-1866
28S-1871-1889
28S-2350-2369
28S-2374-2419
28S-2412-2437
28S-2493-2526
28S-2739-2764
28S-2789-2816
28S-2815-2835
28S-2834-2856
28S-2869-2893
28S-2894-2916
28S-3608-3623
28S-3645-3670
28S-3692-3721
28S-3722-3762
28S-3746-3762
28S-3778-3799
28S-4084-4103
28S-4218-4253
28S-4253-4278
28S-4338-4358
28S-4358-4396
28S-4440-4466
28S-4513-4533
28S-4635-4668
28S-4943-4973
28S-4987-5003
28S-5007-5022
5S-2-38
ETS1-3654-3675
ETS2-117-137
ITS2-1167-1204
18S-1-37

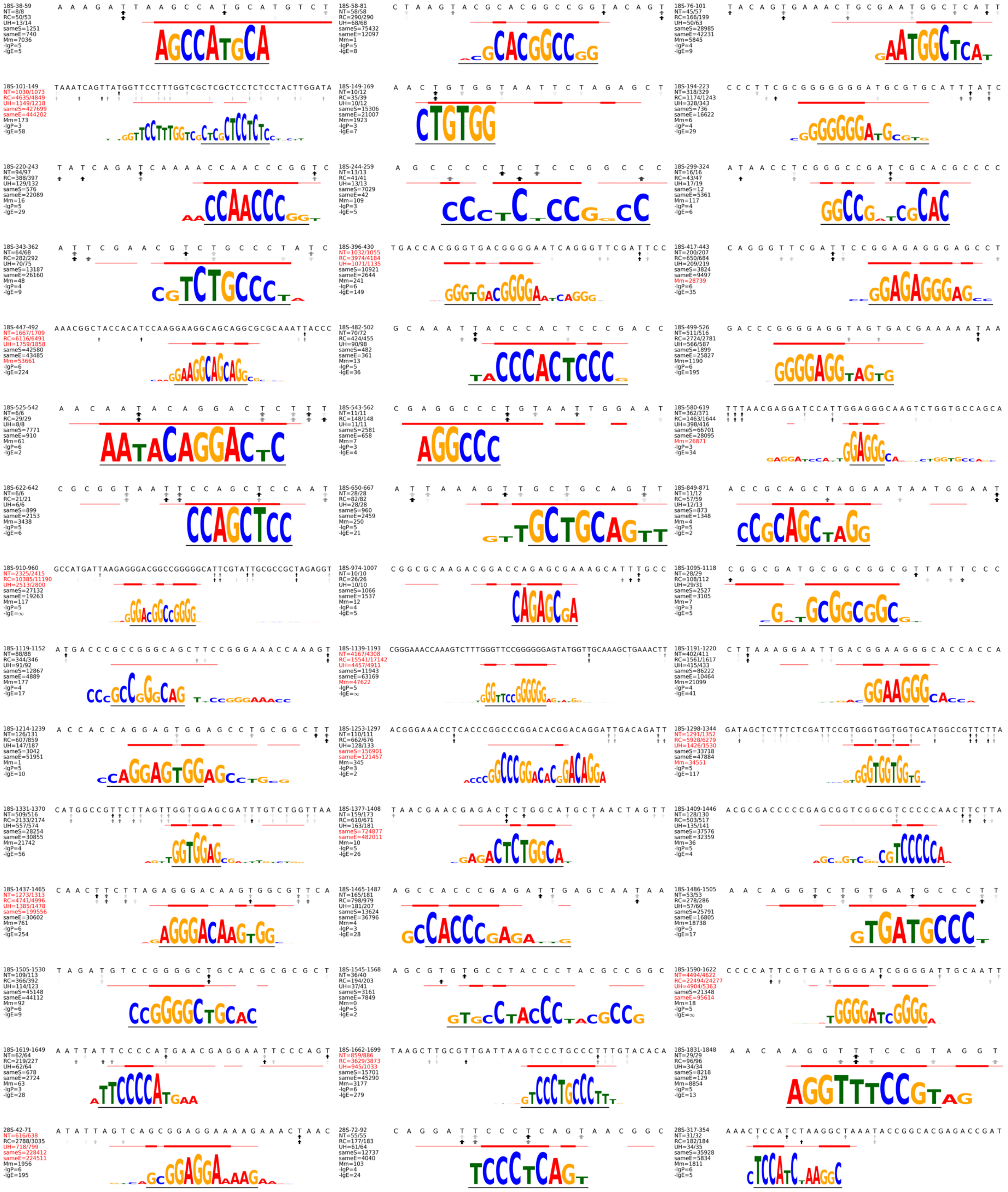

18S-38-59 NT=8/8 RC=50/53 UH=13/14 sameS=1251 sameE=740 Mm=7036 -lgP=5 -lgE=5
AAAGATTAAGCCATGCATGTCT
18S-58-81 NT=58/58 RC=290/290 UH=68/68 sameS=75432 sameE=12097 Mm=1 -lgP=5 -lgE=8
CTAAGTACGCACGGCCGGTACAGT
18S-76-101 NT=45/57 RC=166/199 UH=50/63 sameS=28985 sameE=42231 Mm=5845 -lgP=4 -lgE=9
TACAGTGAAACTGCGAATGGCTCATT
18S-101-149 NT=1030/1073 RC=4635/4849 UH=1149/1218 sameS=427699 sameE=444202 Mm=173 -lgP=3 -lgE=58
TAAATCAGTTATGGTTCCTTTGGTCGCTCGCTCCTCTCCTACTTGGATA
18S-149-169 NT=10/12 RC=35/39 UH=10/12 sameS=15306 sameE=21007 Mm=1923 -lgP=3 -lgE=7
AACTGTGGTAATTCTAGAGCT
18S-194-223 NT=318/329 RC=1174/1243 UH=328/343 sameS=736 sameE=16622 Mm=6 -lgP=4 -lgE=29
CCCTTCGCGGGGGGGATGCGTGCATTTATC
18S-220-243 NT=94/97 RC=388/397 UH=129/132 sameS=576 sameE=22089 Mm=16 -lgP=5 -lgE=29
TATCAGATCAAAACCAACCCGGTC
18S-244-259 NT=13/13 RC=41/41 UH=13/13 sameS=7029 sameE=42 Mm=109 -lgP=3 -lgE=5
AGCCCCTCTCCGGCCC
18S-299-324 NT=16/16 RC=43/47 UH=17/19 sameS=12 sameE=5361 Mm=117 -lgP=4 -lgE=6
ATAACCTCGGGCCGATCGCACGCCCC
18S-343-362 NT=64/68 RC=282/292 UH=70/75 sameS=13187 sameE=26160 Mm=48 -lgP=4 -lgE=9
ATTCGAACGTCTGCCCTATC
18S-396-430 NT=1032/1055 RC=3974/4184 UH=1071/1135 sameS=10921 sameE=2644 Mm=241 -lgP=6 -lgE=149
TGACCACGGGTGACGGGGAATCAGGGTTCGATTCC
18S-417-443 NT=200/207 RC=650/684 UH=209/219 sameS=3824 sameE=9497 Mm=28739 -lgP=6 -lgE=35
CAGGGTTCGATTCCGGAGAGGGAGCCT
18S-447-492 NT=1667/1709 RC=6116/6491 UH=1759/1858 sameS=42580 sameE=43485 Mm=53661 -lgP=6 -lgE=224
AAACGGCTACCACATCCAAGGAAGGCAGCAGGCGCGCAAATTACCC
18S-482-502 NT=70/72 RC=424/455 UH=90/98 sameS=482 sameE=361 Mm=13 -lgP=5 -lgE=36
GCAAATTACCCACTCCCGACC
18S-499-526 NT=511/516 RC=2724/2781 UH=566/587 sameS=1899 sameE=25827 Mm=1190 -lgP=6 -lgE=195
GACCCGGGGAGGTAGTGACGAAAAATAA
18S-525-542 NT=6/6 RC=29/29 UH=8/8 sameS=7771 sameE=910 Mm=61 -lgP=6 -lgE=2
AACAATACAGGACTCTTT
18S-543-562 NT=11/11 RC=148/148 UH=11/11 sameS=2581 sameE=658 Mm=7 -lgP=3 -lgE=4
CGAGGCCCTGTAATTGGAAT
18S-580-619 NT=362/371 RC=1463/1644 UH=398/416 sameS=66701 sameE=28095 Mm=26871 -lgP=3 -lgE=34
TTTAACGAGGATCCATTGGAGGGCAAGTCTGGTGCCAGCA
18S-622-642 NT=6/6 RC=21/21 UH=6/6 sameS=899 sameE=2153 Mm=3438 -lgP=5 -lgE=6
CGCGGTAATTCCAGCTCCAAT
18S-650-667 NT=28/28 RC=82/82 UH=28/28 sameS=960 sameE=2459 Mm=250 -lgP=4 -lgE=21
ATTAAAGTTGCTGCAGTT
18S-849-871 NT=11/12 RC=57/59 UH=12/13 sameS=873 sameE=1348 Mm=4 -lgP=5 -lgE=2
ACCGCAGCTAGGAATAATGGAAT
18S-910-960 NT=2325/2415 RC=10385/11190 UH=2513/2800 sameS=27132 sameE=19263 Mm=117 -lgP=5 -lgE=∞
GCCATGATTAAGAGGGACGGCCGGGGGCATTCGTATTGCGCCGCTAGAGGT
18S-974-1007 NT=10/10 RC=26/26 UH=10/10 sameS=1066 sameE=1537 Mm=12 -lgP=4 -lgE=5
CGGCGCAAGACGGACCAGAGCGAAAGCATTTGCC
18S-1095-1118 NT=28/29 RC=108/112 UH=29/31 sameS=2527 sameE=3105 Mm=7 -lgP=3 -lgE=5
CGGCGATGCGGCGGCGTTATTCCC
18S-1119-1152 NT=88/88 RC=344/346 UH=91/92 sameS=12867 sameE=4889 Mm=177 -lgP=4 -lgE=17
ATGACCCGCCGGGCAGCTTCCGGGAAACCAAAGT
18S-1139-1193 NT=4167/4308 RC=15541/17142 UH=4457/4911 sameS=11943 sameE=63169 Mm=47622 -lgP=5 -lgE=∞
CGGGAAACCAAAGTCTTTGGGTTCCGGGGGGAGTATGGTTGCAAAGCTGAAACTT
18S-1191-1220 NT=402/411 RC=1561/1617 UH=415/433 sameS=86222 sameE=10464 Mm=21099 -lgP=4 -lgE=41
CTTAAAGGAATTGACGGAAGGGCACCACCA
18S-1214-1239 NT=126/131 RC=607/859 UH=147/187 sameS=3042 sameE=51951 Mm=1 -lgP=5 -lgE=10
ACCACCAGGAGTGGAGCCTGCGGCTT
18S-1253-1297 NT=110/111 RC=662/676 UH=128/133 sameS=156901 sameE=121457 Mm=345 -lgP=3 -lgE=2
ACGGGAAACCTCACCCGGCCCGGACACGGACAGGATTGACAGATT
18S-1298-1344 NT=1291/1352 RC=5928/6279 UH=1426/1530 sameS=33718 sameE=47884 Mm=34551 -lgP=5 -lgE=117
GATAGCTCTTTCTCGATTCCGTGGGTGGTGGTGCATGGCCGTTCTTA
18S-1331-1370 NT=509/516 RC=2133/2174 UH=557/574 sameS=28254 sameE=30855 Mm=21742 -lgP=4 -lgE=56
CATGGCCGTTCTTAGTTGGTGGAGCGATTTGTCTGGTTAA
18S-1377-1408 NT=159/173 RC=610/671 UH=163/181 sameS=724877 sameE=482011 Mm=10 -lgP=5 -lgE=26
TAACGAACGAGACTCTGGCATGCTAACTAGTT
18S-1409-1446 NT=128/130 RC=503/517 UH=135/141 sameS=37576 sameE=32359 Mm=36 -lgP=5 -lgE=4
ACGCGACCCCCGAGCGGTCGGCGTCCCCCAACTTCTTA
18S-1437-1465 NT=1273/1313 RC=4741/4996 UH=1385/1478 sameS=199556 sameE=30602 Mm=761 -lgP=6 -lgE=254
CAACTTCTTAGAGGGACAAGTGGCGTTCA
18S-1465-1487 NT=165/181 RC=798/979 UH=181/207 sameS=13624 sameE=36796 Mm=4 -lgP=3 -lgE=28
AGCCACCCGAGATTGAGCAATAA
18S-1486-1505 NT=53/53 RC=278/286 UH=57/60 sameS=25791 sameE=16805 Mm=18738 -lgP=5 -lgE=17
AACAGGTCTGTGATGCCCTT
18S-1505-1530 NT=109/113 RC=366/392 UH=114/123 sameS=45148 sameE=44112 Mm=92 -lgP=6 -lgE=9
TAGATGTCCGGGGCTGCACGCGCGCT
18S-1545-1568 NT=36/40 RC=194/203 UH=37/41 sameS=3161 sameE=7849 Mm=0 -lgP=5 -lgE=2
AGCGTGTGCCTACCCTACGCCGGC
18S-1590-1622 NT=4494/4622 RC=22494/24277 UH=4904/5363 sameS=21348 sameE=95614 Mm=18 -lgP=5 -lgE=∞
CCCCATTCGTGATGGGGATCGGGGATTGCAATT
18S-1619-1649 NT=62/64 RC=219/227 UH=62/64 sameS=678 sameE=2724 Mm=63 -lgP=3 -lgE=28
AATTATTCCCCATGAACGAGGAATTCCCAGT
18S-1662-1699 NT=859/886 RC=3629/3873 UH=945/1033 sameS=15701 sameE=45290 Mm=3177 -lgP=6 -lgE=279
TAAGCTTGCGTTGATTAAGTCCCTGCCCTTTGTACACA
18S-1831-1848 NT=29/29 RC=96/96 UH=34/34 sameS=8218 sameE=129 Mm=8854 -lgP=5 -lgE=13
AACAGGTTTCCGTAGGT
28S-42-71 NT=616/638 RC=2788/3035 UH=718/799 sameS=228412 sameE=224511 Mm=1956 -lgP=6 -lgE=195
ATATTAGTCAGCGGAGGAAAAGAAACTAAC
28S-72-92 NT=55/55 RC=177/183 UH=61/64 sameS=12737 sameE=4040 Mm=103 -lgP=4 -lgE=24
CAGGATTCCCTCAGTAACGGC
28S-317-354 NT=31/32 RC=182/184 UH=34/35 sameS=35928 sameE=5834 Mm=1811 -lgP=6 -lgE=5
AAACTCCATCTAAGGCTAAATACCGGCACGAGACCGAT

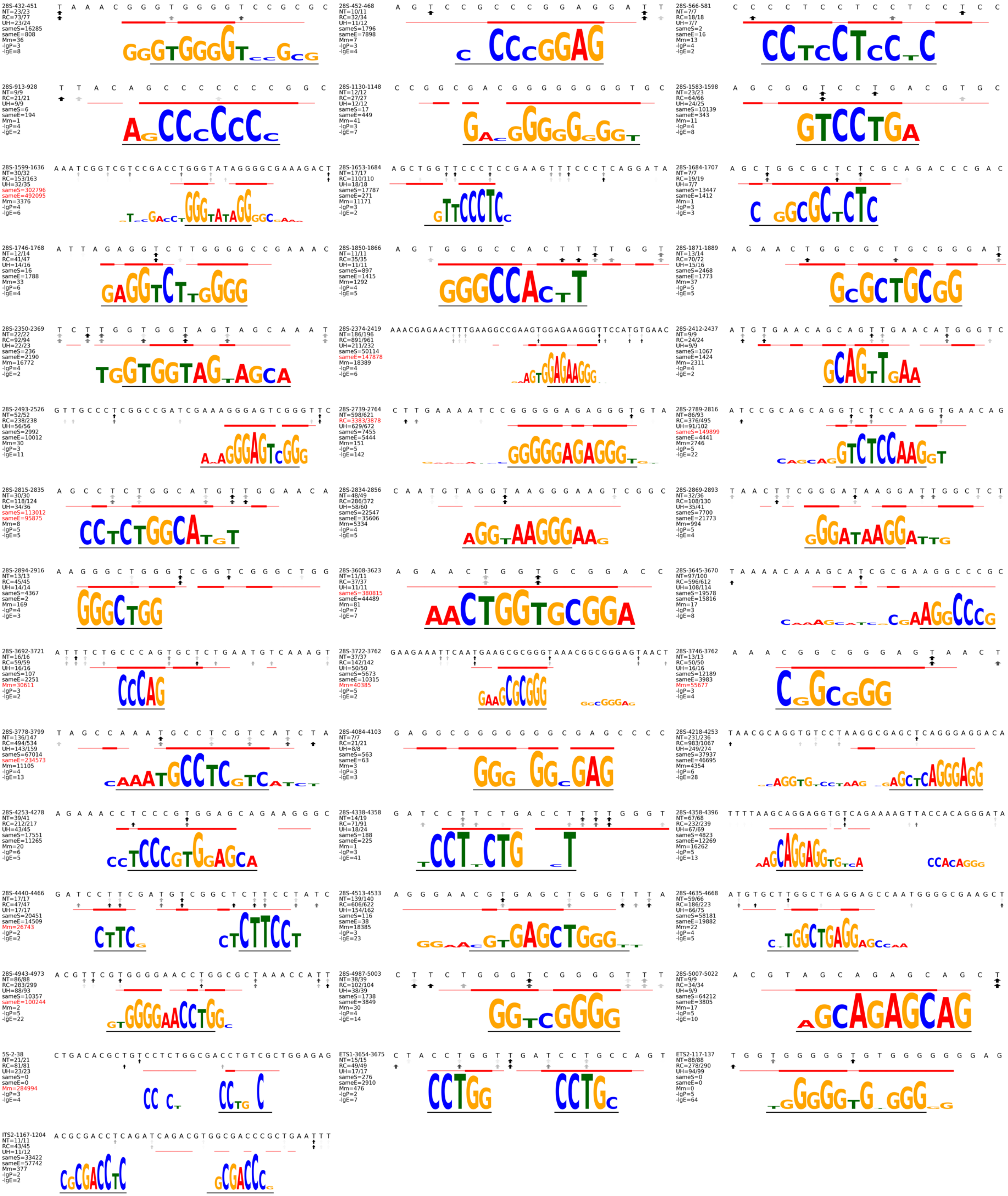
28S-432-451
28S-452-468
28S-566-581
28S-913-928
28S-1130-1148
28S-1583-1598
28S-1599-1636
28S-1653-1684
28S-1684-1707
28S-1746-1768
28S-1850-1866
28S-1871-1889
28S-2350-2369
28S-2374-2419
28S-2412-2437
28S-2493-2526
28S-2739-2764
28S-2789-2816
28S-2815-2835
28S-2834-2856
28S-2869-2893
28S-2894-2916
28S-3608-3623
28S-3645-3670
28S-3692-3721
28S-3722-3762
28S-3746-3762
28S-3778-3799
28S-4084-4103
28S-4218-4253
28S-4253-4278
28S-4338-4358
28S-4358-4396
28S-4440-4466
28S-4513-4533
28S-4635-4668
28S-4943-4973
28S-4987-5003
28S-5007-5022
5S-2-38
ETS1-3654-3675
ETS2-117-137
ITS2-1167-1204
