# Supplementary material for: Computational meta-analysis of ribosomal RNA fragments: potential targets and interaction mechanisms
Source: Nucleic Acids Res. 2021 Mar 27;49(7):4085–103. doi: 10.1093/nar/gkab190 (PMC8053083; doi:10.1093/nar/gkab190)
Supplement: gkab190_Supplemental_Files [file gkab190_supplemental_files.zip › guan-nar-supp.pdf]

Supplementary information

Table S1. Statistics of CLASH reads containing rRFs, miRNAs and tRFs (see Excel file).

Table S2. Number of rRFs covering expansion segments (see Excel file).

Table S3. Top 20 interactions between rRFs and different types of target genes in forward and reverse CLASH pairs (see Excel file).

Table S4. rRFs interacting with the boundaries of known agotrons (see Excel file).

Table S5. Novel agotron candidates found in chimeras with rRFs (see Excel file).

Table S6. List of rRFs with a single (>95% of all hybrid reads) target gene (see Excel file).

Table S7. Enrichments of Gene Ontology terms and KEGG pathways in high-confidence protein coding targets (see Excel file).

Table S8. Frequency of several rRF isoforms in different datasets in reads per million (RPM) and in relative abundance (RA) among fragments of a given rRNA (see Excel file).

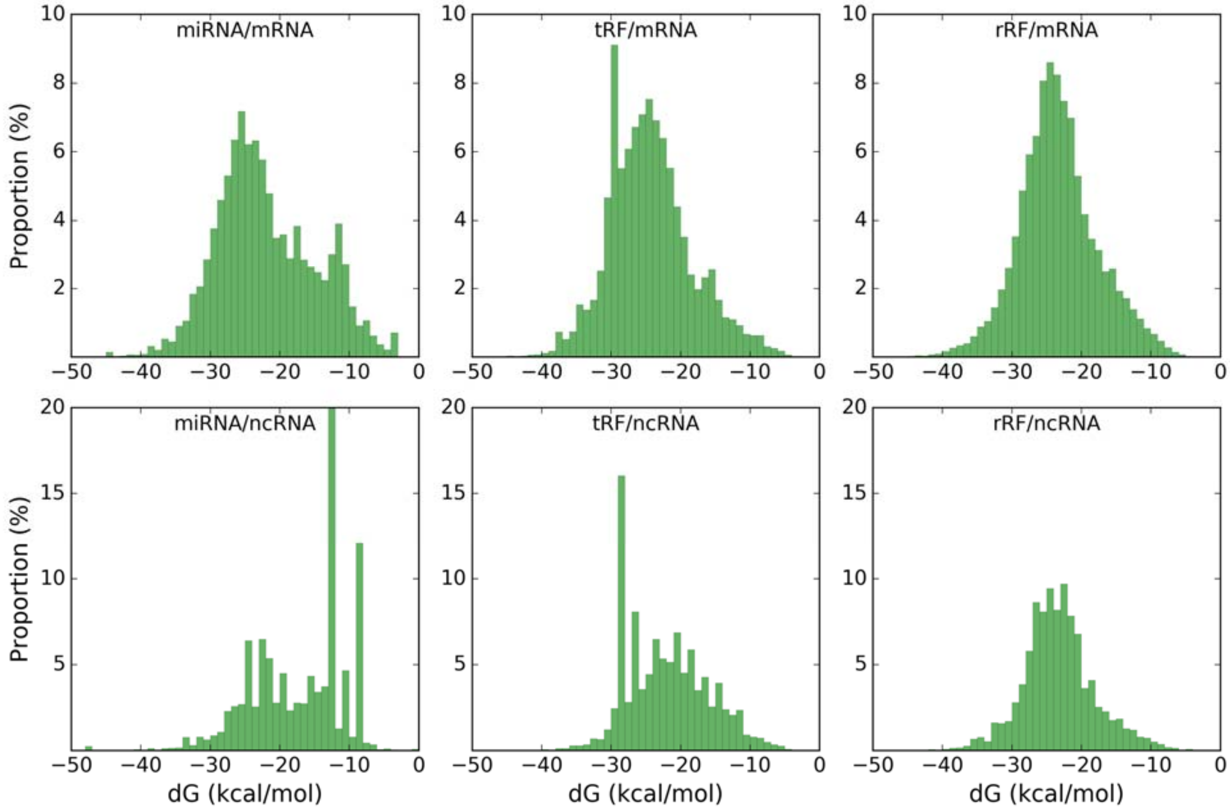

Figure S1. MFE distributions indicated as "guide/target" for various types of sRNA guides bound to their targets identified as pairs in CLASH chimeric reads. Unique chimeras are shown.

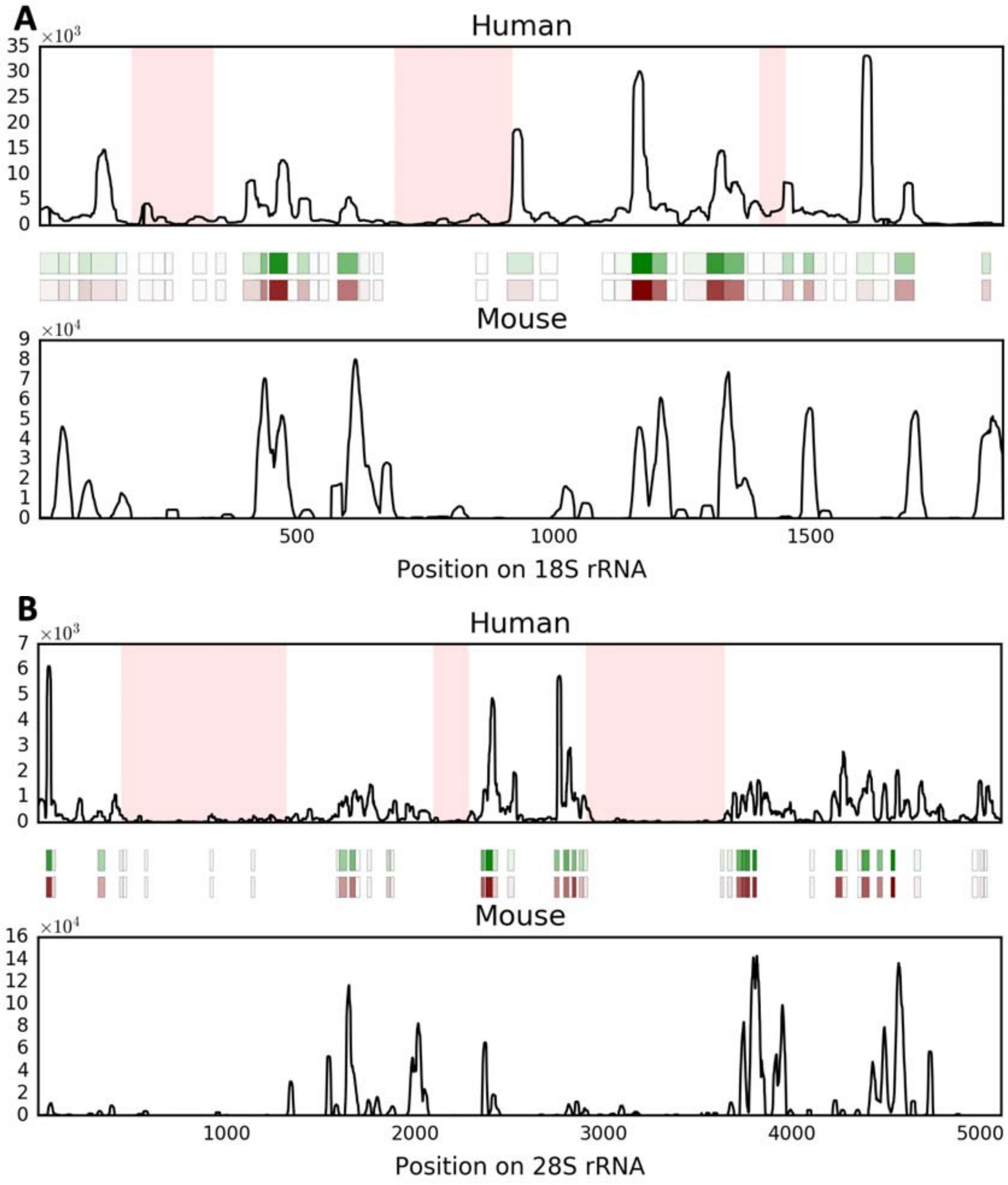

Figure S2. Origin of rRFs targeting the protein coding genes on (A) 18S rRNA and (B) 28S rRNA detected in human Ago1 CLASH datasets (top) and mouse Ago2 PAR-CLIP datasets (bottom), with rRF RPM on the y-axis. Pale red areas are large human expansion segments including (from left to right): ES3S, ES6S and ES9S in 18S, ES7L, ES15L and ES27L in 28S. Colored boxes indicate the groups of identified motifs (Fig. S8). The color intensity shows the geometric mean of frequencies of group-specific human rRFs and corresponding mouse rRFs, the latter detected in cytoplasm (green) or nucleus (red), respectively.

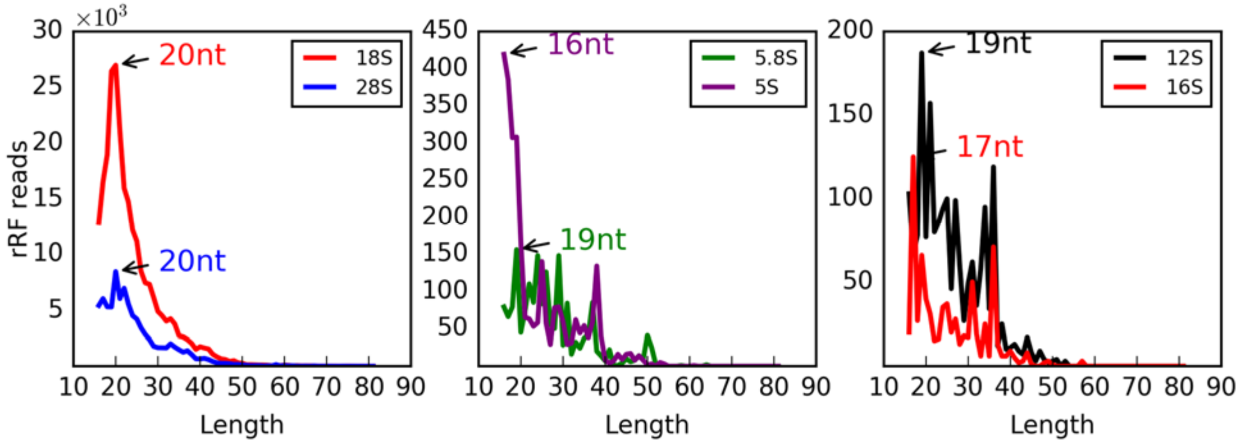

Figure S3. Length distribution (by host rRNA) of CLASH rRFs targeting protein coding genes.

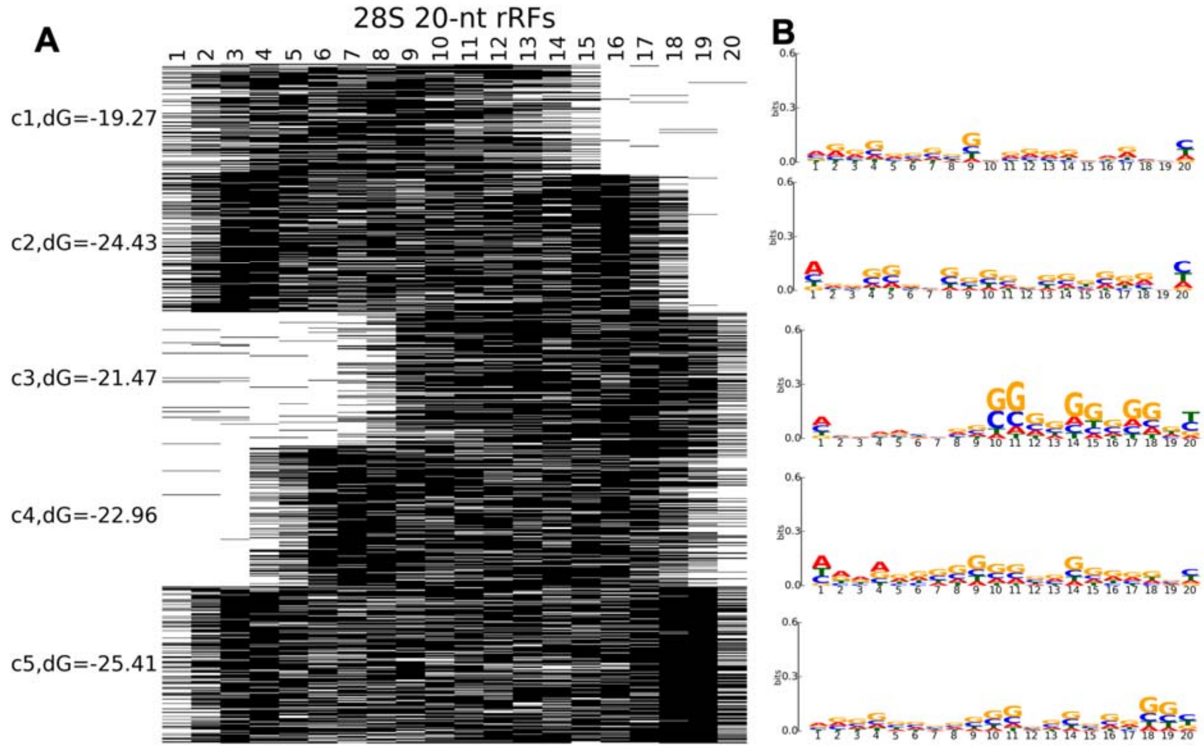

Figure S4. Clustering of 20-nt rRFs from 28S rRNAs in CLASH. (A) Base-pairing patterns of 20-nt rRFs in unique rRF/target interactions. (B) Sequence logos show the nucleotide composition patterns for unique rRF isoforms in every cluster.

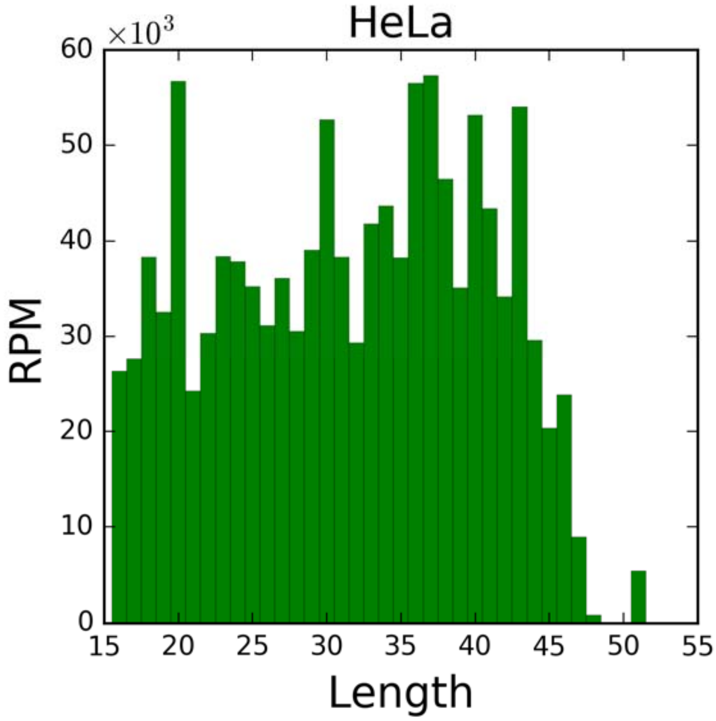

Figure S5. Length distribution of cprRFs.

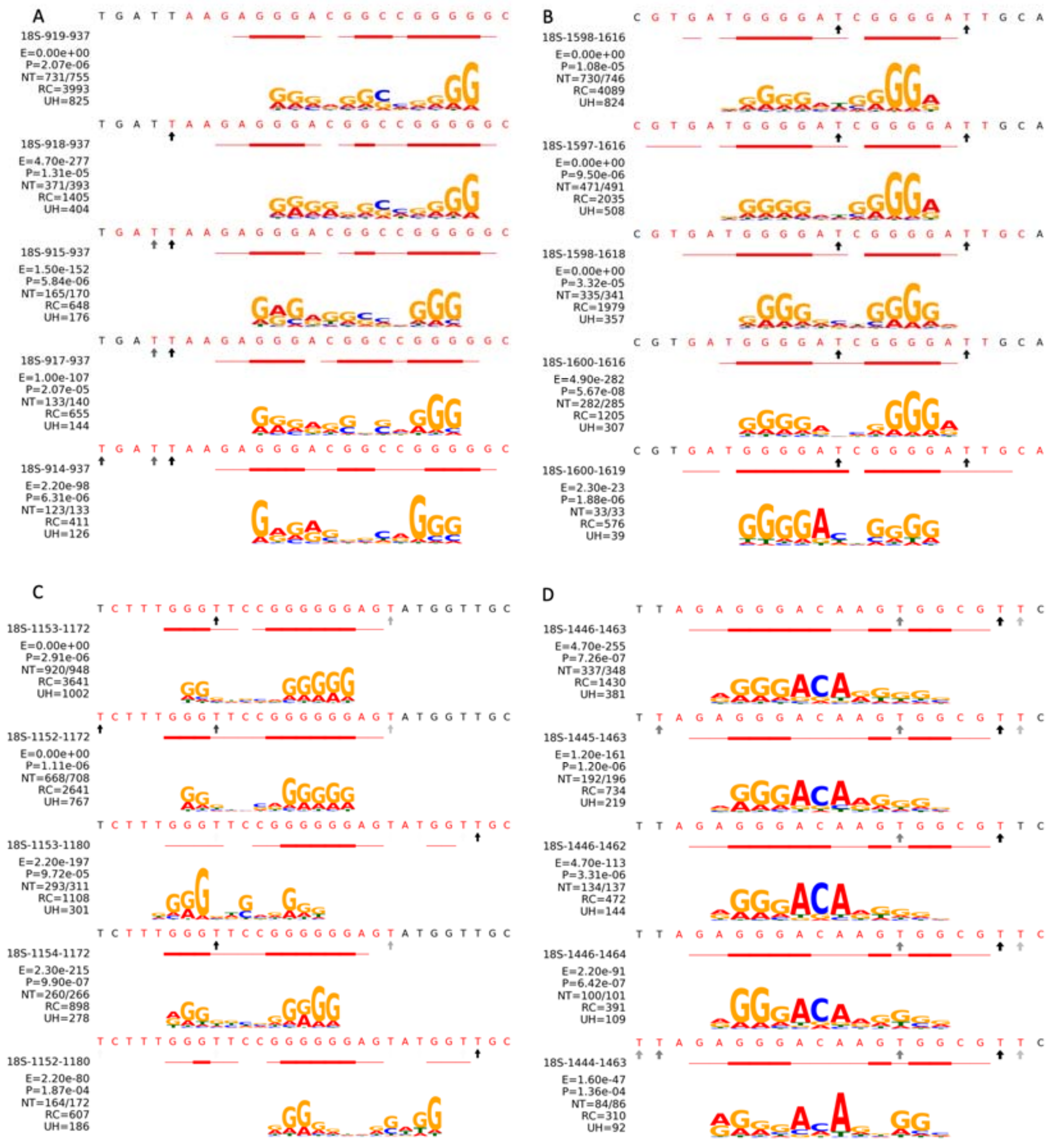

Figure S6. Hybridization patterns, target motifs and secondary structures, and crosslinking of CLASH rRF (A) 18S-919-937, (B) 18S-1598-1616, (C) 18S-1153-1172 (D) 18S-1446-1464 and four other rRFs with at least 300 supporting reads overlapping them. The longest union sequence of overlapping rRFs is shown on the top. Sequence of each individual rRF is in red. Motifs found by MEME among the unique targets of each rRF isoform were aligned to the rRF sequence by FIMO and a reverse complement of each motif logo is shown. The RNAhybrid line shows hybridizing rRF/target nucleotides (thick lines in ≥80%, and thin lines in ≥50% of the targets). Arrows show T→C conversions identified in PAR-CLIP datasets with frequencies depicted as varying from black (highest RPM) to gray (lower RPM).

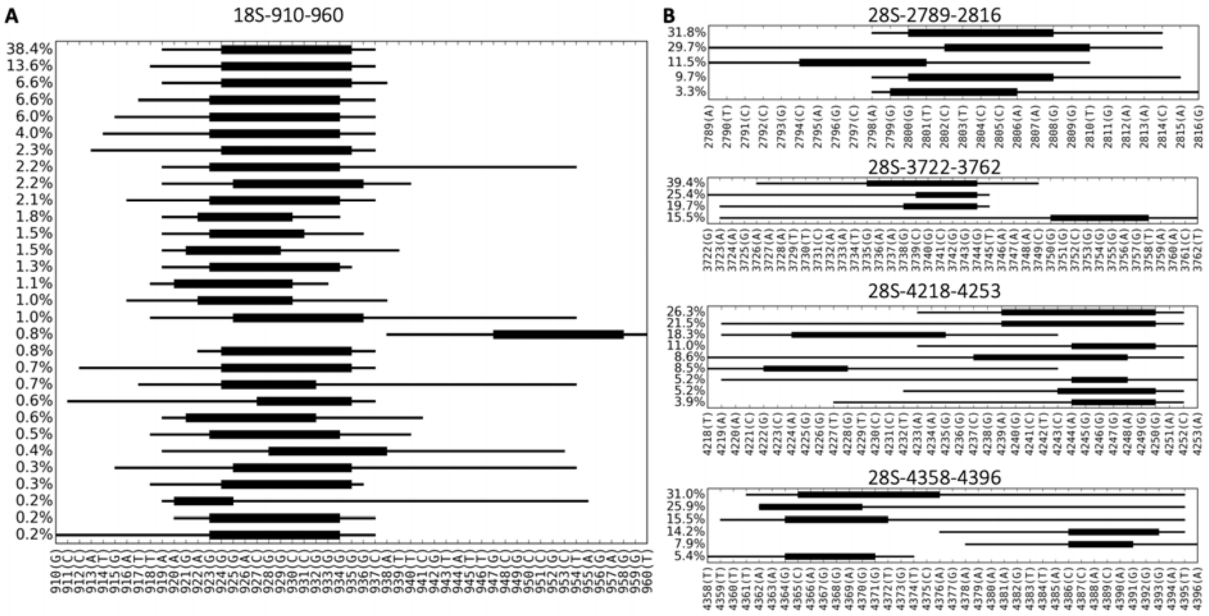

Figure S7. Arrangement of rRFs and motifs in groups. Thin lines indicate the coordinates of rRFs and thick lines indicate the coordinates of motifs. Relative frequencies of rRFs in every group are shown on y-axis.

Figure S8. (***See pdf file***) Hybridization patterns in rRFs groups (see PDF file). The nucleotide frequencies in target motifs of all rRF isoforms are combined for every position in the group. The relative frequency of the nucleotide matched to the rRF sequence is shown in the logo. The height of the letters is scaled to the number of reads supporting the motifs for every position. The red line shows hybridizing rRF/target nucleotides (thick lines in >80%, and thin lines in >50% of the targets). Black line underlines the motif of the most abundant rRF isoform and negative logarithms of its e-value (MEME) and p-value (FIMO) are printed on the left. Two lines of arrows show the T→C conversions identified in human (top) and mouse (bottom) PAR-CLIP datasets with frequencies depicted as varying from black (highest RPM) to gray (lower RPM). NT: number of unique target genes. RC: total number of CLASH reads. UH: unique rRF/target hybrids in CLASH. sameS: total frequencies of cprRFs with the same start to every motif-containing rRF in the group. sameE: total frequencies of cprRFs with the same end to every motif-containing rRF in the group. Mm: frequencies of mouse Ago2 rRFs aligned within the group. The top 20 values in each category are shown in red.

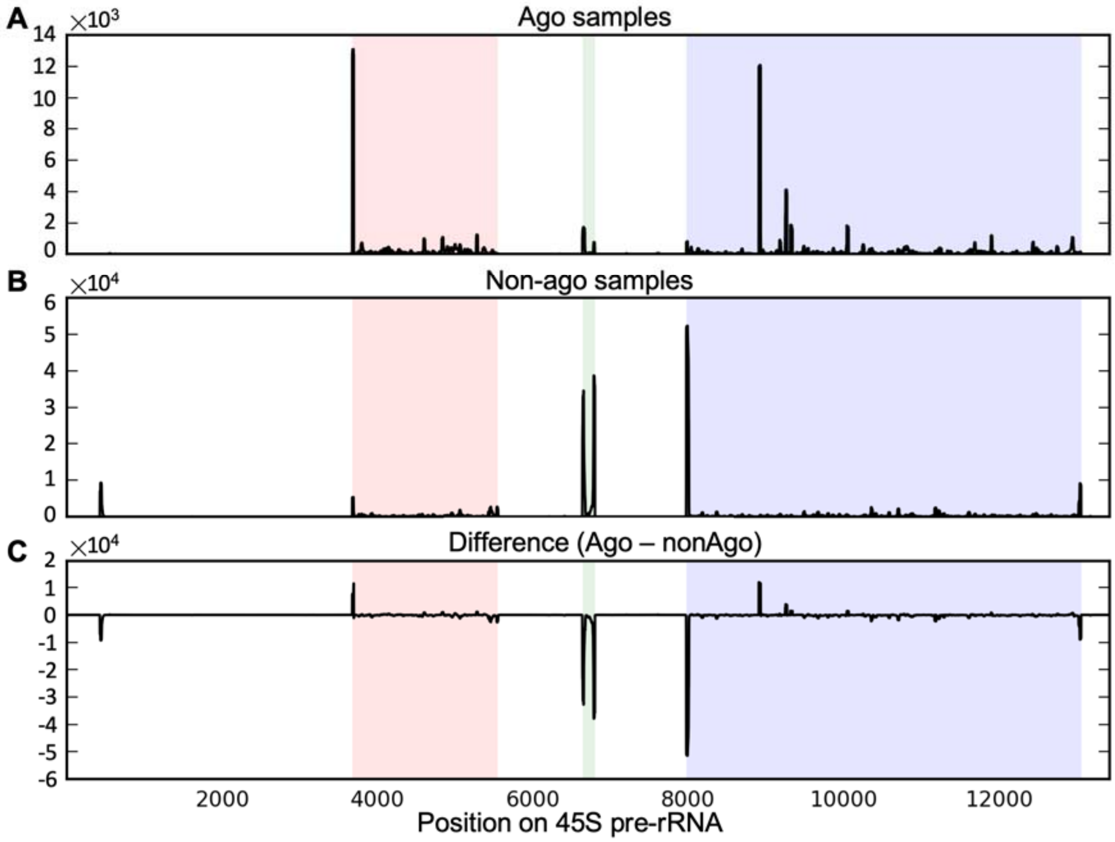

Figure S9. Coverages on 45S pre-rRNA by rRFs identified in (A) Ago-IP samples and (B) non-Ago-IP samples described in Table S9. The coverages for a given position is normalized by the total number of 45S rRFs in each sample and then are averaged in two groups. (C) The differences of averaged relative coverages between Ago and non-Ago identified rRFs.
